# Protocol for the development of a core outcome set for stillbirth care research (iCHOOSE Study)

**DOI:** 10.1136/bmjopen-2021-056629

**Published:** 2022-02-09

**Authors:** Danya Bakhbakhi, Abigail Fraser, Dimitris Siasakos, Lisa Hinton, Anna Davies, Abi Merriel, James M N Duffy, Maggie Redshaw, Mary Lynch, Laura Timlin, Vicki Flenady, Alexander Edward Heazell, Soo Downe, Pauline Slade, Sara Brookes, Aleena Wojcieszek, Margaret Murphy, Heloisa de Oliveira Salgado, Danielle Pollock, Neelam Aggarwal, Irene Attachie, Susannah Leisher, Wanijiru Kihusa, Kate Mulley, Lindsey Wimmer, UK iCHOOSE parent involvement group, Christy Burden

**Affiliations:** 1 Translational Health Sciences, University of Bristol Medical School, Bristol, UK; 2 Population Health Sciences, University of Bristol Medical School, Bristol, UK; 3 UCL EGA Institute for Women's Health, UCL, London, UK; 4 The Healthcare Improvement Studies Institute, University of Cambridge, Cambridge, UK; 5 Centre for Academic Child Health, University of Bristol, Bristol, UK; 6 Obstetrics and Gynaecology Department, North Middlesex University Hospital NHS Trust, London, UK; 7 Department of Population Health, NPEU, Oxford, UK; 8 Faculty of Health Sciences, University of Bristol, Bristol, UK; 9 Women & Children's Health Department, North Bristol NHS Trust, Bristol, UK; 10 Centre of Research Excellence in Stillbirth, Mater Research Institute-University of Queensland, Brisbane, Queensland, Australia; 11 Maternal and Fetal Health Research Centre, University of Manchester, Manchester, UK; 12 Research in Childbirth and Health, University of Central Lancashire, Preston, UK; 13 Psychological Sciences, University of Liverpool, Liverpool, UK; 14 Institute of Cancer and Genomic Sciences, University of Birmingham, Birmingham, UK; 15 Nursing and Midwifery, University College Cork National University of Ireland, Cork, Ireland; 16 Department of Social Medicine, University of São Paulo, Sao Paulo, Brazil; 17 Public Health, The University of Adelaide, Adelaide, South Australia, Australia; 18 Department of Obstetrics and Gynaecology, Post Graduate Institute of Medical Education and Research, Chandigarh, India; 19 Department of Nursing and Midwifery, University of Health and Allied Sciences School of Public Health, Hohoe, Ghana; 20 International Stillbirth Alliance, Millburn, New Jersey, USA; 21 Still a mum, Nairobi, Kenya; 22 Research department, Sands, London, UK; 23 Star Legacy Foundation, Minneapolis, Minnesota, USA

**Keywords:** obstetrics, qualitative research, quality in health care, protocols & guidelines, fetal medicine, maternal medicine

## Abstract

**Introduction:**

Stillbirth is associated with significant physical, psychosocial and economic consequences for parents, families, wider society and the healthcare system. There is emerging momentum to design and evaluate interventions for care after stillbirth and in subsequent pregnancies. However, there is insufficient evidence to inform clinical practice compounded by inconsistent outcome reporting in research studies. To address this paucity of evidence, we plan to develop a core outcome set for stillbirth care research, through an international consensus process with key stakeholders including parents, healthcare professionals and researchers.

**Methods and analysis:**

The development of this core outcome set will be divided into five distinct phases: (1) Identifying potential outcomes from a mixed-methods systematic review and analysis of interviews with parents who have experienced stillbirth; (2) Creating a comprehensive outcome long-list and piloting of a Delphi questionnaire using think-aloud interviews; (3) Choosing the most important outcomes by conducting an international two-round Delphi survey including high-income, middle-income and low-income countries; (4) Deciding the core outcome set by consensus meetings with key stakeholders and (5) Dissemination and promotion of the core outcome set. A parent and public involvement panel and international steering committee has been convened to coproduce every stage of the development of this core outcome set.

**Ethics and dissemination:**

Ethical approval for the qualitative interviews has been approved by Berkshire Ethics Committee REC Reference 12/SC/0495. Ethical approval for the think-aloud interviews, Delphi survey and consensus meetings has been awarded from the University of Bristol Faculty of Health Sciences Research Ethics Committee (Reference number: 116535). The dissemination strategy is being developed with the parent and public involvement panel and steering committee. Results will be published in peer-reviewed specialty journals, shared at national and international conferences and promoted through parent organisations and charities.

**PROSPERO registration number:**

CRD42018087748.

**Strengths and limitations of this study**

- Using robust and transparent methodology, this will be the first core outcome set developed for use in stillbirth care research, which will ultimately improve evidence synthesis in this field and could reduce research wastage.
- In-depth qualitative interviews with parents will enable the identification of novel and parent-important outcomes not identified from the systematic review.
- Parent representation is a strength of this study; we are including bereaved parent stakeholders at every stage of the development, coproducing the research with a parent involvement panel, and have international parent representation within the project steering committee.
- Qualitative interviews (in stage 1) include UK parents only, however, to help mitigate this limitation and increase the generalisability of the results, we are triangulating our findings with outcomes identified in the systematic review of global literature along with recuiting international stakeholders for the think-aloud interviews, Delphi survey and consensus meetings.
- Due to funding limitations and translation costs, the Delphi survey and consensus meetings will be conducted in the English language only, however, future research will endeavour to validate the core outcome set in languages other than English.

## Introduction

Worldwide it is estimated that there are 2 million stillbirths every year.^1^ Stillbirth is associated with significant physical, psychosocial, health and economic costs for parents, their families, wider society and the healthcare system.^2–4^ In a subsequent pregnancy, a history of stillbirth has been shown to be associated with higher frequencies of adverse clinical outcomes, including increased risk of stillbirth recurrence, antenatal complications, mental health concerns and impact on subsequent children.^5–8^ The negative consequences of stillbirth are widespread and long-lasting; therefore, it is important to invest in high-quality research to enable healthcare professionals and researchers to deliver the best care for affected families.

Several care-related interventions are available to minimise the negative impact of stillbirth. These interventions can be implemented from the immediate identification of a stillbirth to when parents are discharge from hospital to the community or in a subsequent pregnancy. Examples include, supporting parents’ choices around birth and afterwards, offering opportunities for parents to make memories with their baby, support with postmortem investigation decision making, engagement of the parents in the perinatal mortality review process,^9–12^ bereavement care from healthcare professionals,^13^ counselling and specialist care in subsequent pregnancies.^14^ Yet very little is known about the effectiveness of these interventions.^15^

There is momentum to research, design and evaluate interventions to improve care for parents following stillbirth and in any subsequent pregnancies.^15–17^ However, systematic reviews suggest few methodologically rigorous studies exist to inform clinical practice and their results cannot be synthesised quantitatively due to a high degree of heterogeneity of outcome reporting.^15 17 18^ In 2018, a Cochrane review on care prior to and during subsequent pregnancies following stillbirth for improving outcomes, found insufficient and inconsistent evidence to inform clinical practice.^17^ The authors of this review concluded that it is important to have consistency in data collection across all future trials and this may be facilitated by a core outcome set for stillbirth care research.^17^

A core outcome set is a consensus-derived minimum set of outcomes that should be measured and reported in all research studies of a specific disease or trial population.^19^ It does not preclude the measurement of additional specific outcomes; however, a minimum set of outcomes will allow higher quality of evidence to identify the most effective interventions and care packages offered. A recent web-based survey of healthcare professionals, researchers and advocates identified the development of a core outcomes set for stillbirth (and recurrent stillbirth) research as one of the top five priority research topics to inform clinical practice for the care of families following stillbirth.^20^ Currently, there are no available core outcome sets published for stillbirth care research (ie, research focusing on care after a stillbirth is identified) https://wwwcomet-initiativeorg/Studies.

The inclusion of patients in the development of a core outcome set is paramount as they are the key stakeholders in the research outcomes. Inclusion of parents can lead to a widening of the research agenda, identifying important patient reported outcomes and recognising previously neglected patient outcomes that matter to those who experience stillbirth.^21^ There is a need to develop and evaluate evidence-based interventions using outcomes that directly relate to bereaved parents’ experiences. To enable this, it is essential to establish a minimum set of outcomes that includes parents and relevant stakeholders in the development process. If applied in clinical trials, a core outcome set for stillbirth care research developed with stakeholder input, will provide a tool to give consistency in outcome measurement, minimise reporting bias and allow for direct comparison of interventions and care across research studies. This could lead to better evidence being produced to improve clinical decision making in the future.

## Aim and objectives

### Aim

The International Collaboration for Harmonising Outcomes fOr Stillbirth research and carE (iCHOOSE) study aims to develop a minimum set of outcomes that should be evaluated and reported in all future stillbirth care research in high-income, middle-income and low-income country settings, through an international consensus process of key stakeholders including parents, healthcare professionals, researchers and charity representatives.

### Objectives

1. To investigate what outcomes are reported in existing studies assessing the impact of stillbirth on parents.
2. To investigate parental experiences following stillbirth and identify important outcomes for bereaved parents not reported in the scientific literature.
3. To pilot and develop a Delphi questionnaire, using think-aloud interviews.
4. To achieve international consensus on a core outcome set for stillbirth care research using a Delphi survey technique and stakeholder consensus meetings.
5. To disseminate and promote the core outcome set for stillbirth care research.

## Methods and analysis

There is no standardised way to develop a core outcome set.^21^ The Core Outcome Measures in Effectiveness Trials (COMET) initiative has collated methodological resources to assist with the development of the COS including a systematic review outlining the issues to consider.^21–24^ COMET resources, including the COMET Handbook: V.1.0 and reviewed published core outcome sets have been used to inform the study design.^23–30^ This study is prospectively registered on the COMET website https://wwwcomet-initiativeorg/studies/details/775. The Core Outcome Set-STAndards for Development (COS-STAD) and the COS-STAndardised Protocol Items (COS-STAP) have been followed in the planning of the methods of this core outcome set project.^31 32^ See online supplemental material 1: COS-STAndardised Protocol Items (COS-STAP) Checklist for the iCHOOSE Study. The final core outcome set will be reported in accordance with the COS-STAndards for Reporting statement (COS-STAR).^33^

10.1136/bmjopen-2021-056629.supp1 Supplementary data

### Scope of this core outcome set

#### Health condition and population

The core outcome set will be applicable to families who have experienced a stillbirth in a singleton or multiple pregnancy. We will aim for this core outcome set to be applicable to all countries internationally including high-inome, middle-income and low-income countries. The definition of stillbirth varies internationally and therefore the gestation will be dependent on the study setting. It is our intention that this core outcome set could be applied to stillbirths from at least 20 weeks’ gestation, including antepartum and intrapartum stillbirths from any cause including due to a congenital abnormality. We will set exclude outcomes related to the termination of pregnancy and neonatal death population.

#### Interventions

**Figure 1:**
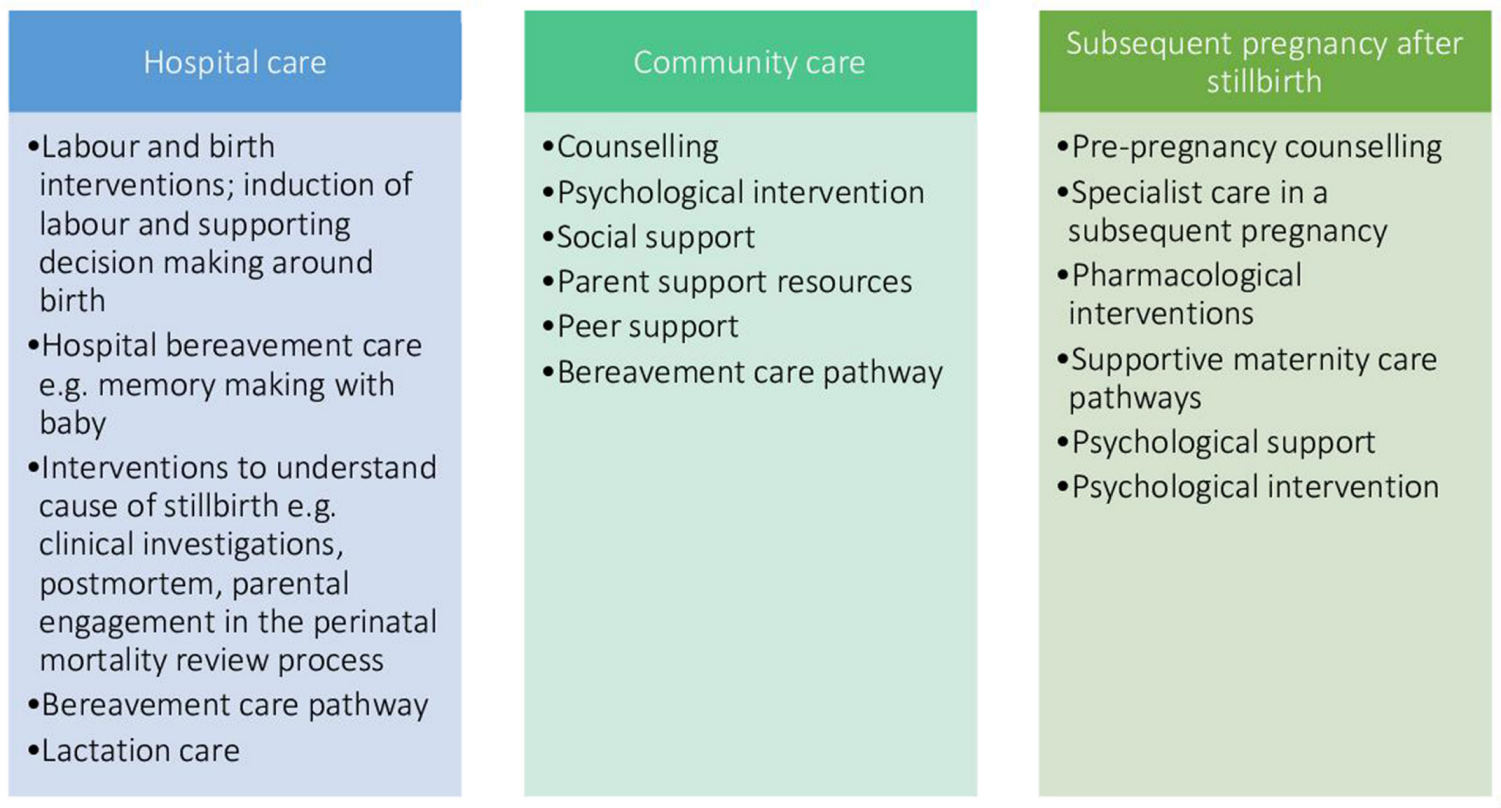
Types of interventions after stillbirth that should be evaluated using outcomes identified in the core outcome set.

The core outcome set will be relevant to all stillbirth care research. Stillbirth care research includes the care that parents (and families) receive after a stillbirth has been identified. The core outcome set will not be limited by the type of intervention or the setting in which it is delivered. It will cover all medical and psychosocial interventions and care parents are offered following a stillbirth and in a subsequent pregnancy.^15 17^ See figure 1: types of interventions after stillbirth that should be evaluated using outcomes identified in the core outcome set.

#### Context

The core outcome set will be developed for use in all stillbirth care research (eg, randomised controlled trials, observational studies and systematic reviews). It is also anticipated that it could be used in the evaluation of clinical practice guidelines, care pathways for bereaved parents and training for healthcare professional.^34^

### Patient and public involvement

Parent perspectives are integral to every stage of the development, including the input into this protocol, the systematic review, qualitative interviews, Delphi survey, consensus meeting and dissemination of results. A parent involvement panel has been established and training is being provided using methods exemplified by the National Institute of Health Research NIHR Centre for Engagement and Dissemination. The parent involvement panel have also co-designed the parent animation video to aid recruitment https://vimeocom/292143259/f2edb109dd.

### Steering Committee

An international expert steering committee including healthcare professionals, parents with a lived experience of stillbirth, charity representatives and researchers with diverse expertise has been convened to guide the research design, recruitment and development of the core outcome set. This group has stakeholder representation from Europe, Australia, North America, South America, Africa and Asia.

### Collaborations

We have established the iCHOOSE initiative. The iCHOOSE collaboration aims to develop a core outcome set for stillbirth care research with the overall aim of improving outcomes for parents and the wider family. This collaboration is endorsed by the Core Outcomes in Women’s Health (CROWN) initiative; the Medical Sociology and Health Experiences Research Group, University of Oxford; the National Stillbirth Centre for Research Excellence, Australia, The Stillbirth and Neonatal Death Charity (Sands); Tommy’s National Centre for Maternity Improvement, Twins Trust, Star Legacy Foundation and International Stillbirth Alliance (ISA).

### Study overview

**Figure 2:**
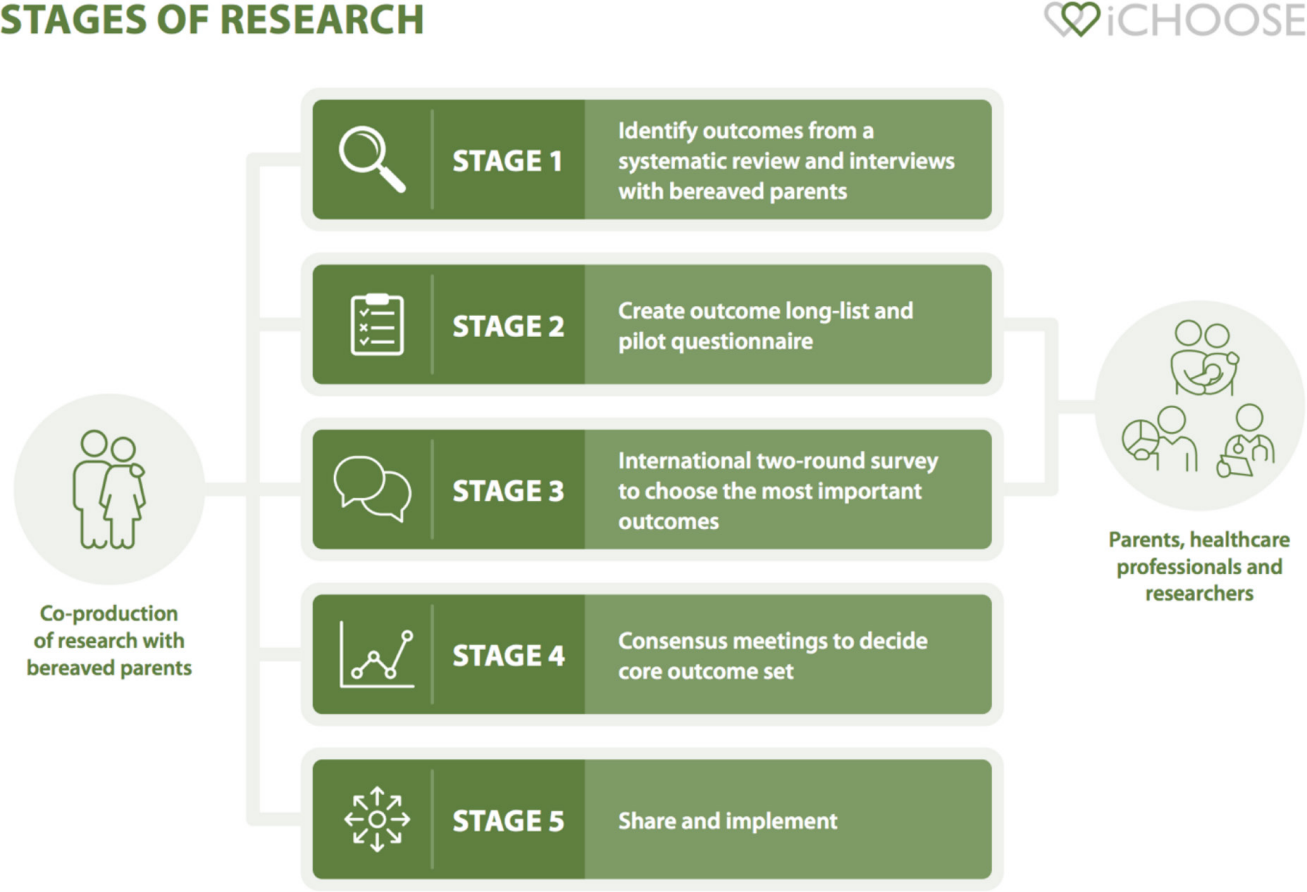
iCHOOSE study overview. iCHOOSE, International Collaboration for Harmonising Outcomes fOr Stillbirth research and carE.

The study will be divided into five distinct stages. See figure 2.

### Stage 1: identifying potential outcomes

#### Systematic review: what outcomes have been reported?

Previously reported outcomes and associated outcome measurement tools relevant to stillbirth care research are being identified through a systematic review of the literature. The electronic databases MEDLINE, PubMed, Embase, Scopus, Amed, BNI, CINAHL, PsycINFO Cochrane Register of Controlled Trials will be searched from 1998 to present. Reference lists of extracted articles will also be searched. We will include all randomised trials, observational and qualitative studies that report an outcome following stillbirth. Case reports, editorials, review articles, abstracts and grey literature will be excluded. Studies including mothers, fathers, children, siblings and grandparents experiencing a stillbirth in a singleton or multiple pregnancy will be included. Studies will not be excluded based on the gestational definition of stillbirth, as the definition varies between jurisdictions. Titles, abstracts and full texts of studies will be screened independently by two review authors using Covidence systematic review software.^35^ Disagreements will be resolved through a third reviewer.

A standardised, prepiloted electronic data extraction form has been developed to extract data. Data will be extracted in duplicate and includes basic publication details (including author and date of publication); study setting; study population; details of intervention (if applicable); study methodology; outcomes measured verbatim, their definition (if stated), their relevant outcome measurement tool (if applicable) and whether the tool is validated for that cultural context and if parents and members of the public were involved in the outcome selection. A sequential explanatory approach will be undertaken, that is, outcomes from quantitative studies will be extracted initially followed by outcomes reported in the qualitative literature. This will be done to compare, and contrast outcomes reported in the qualitative literature. A comprehensive inventory of outcomes reported will be developed from the data extraction The systematic review will be reported using the Preferred Reporting Items for Systematic Reviews and Meta-Analyses guidelines.^36^

#### Qualitative interviews: what outcomes are important to parents?

Capturing patient perspectives is crucial in the development of a core outcome set as they often identify outcomes not considered by other stakeholders or within the literature.^37^ Parents with a lived experience of stillbirth in the United Kingdom (UK) will be recruited to participate in qualitative interviews through Sands, National Health Service Hospital Trusts, the Twins Trust, bereavement support groups, the parent involvement panel and snowballing through personal contacts of the research team and the parent involvement panel. To ensure diverse opinions participants will be purposively sampled for maximum variation. Participants will include mothers and fathers/partners from a wide range of social, ethnic, and cultural backgrounds who have experienced a stillbirth at a range of gestations and time periods since the stillbirth occurred. Parents who have a personal history of a stillbirth at more than 24 weeks’ gestation (UK definition), at least 6 months prior to the study would be eligible to participate. This definition was chosen as recent research has focused on parents’ experiences of care following the death of a baby in pregnancy between 20 and 24 weeks in the UK.^38^ The findings of this research will be incorporated into the systematic review findings. Furthermore, as we are only recruiting UK parents, we plan to triangulate the data with outcomes extracted from the qualitative data from the systematic review. Parents will be interviewed individually or jointly, according to preference. The number of parents recruited will depend on when theoretical saturation is reached (ie, when no new themes emerge).^39^

With informed consent, semistructured interviews with parents will be conducted in either parents’ homes, a suitable private location of their choice or via Zoom teleconference software. A researcher with training in qualitative interview methods will conduct the interviews (DB) supported by an experienced qualitative researcher (LH). The interviews will invite parents to narrate their lived experienced of stillbirth. However, an interview topic guide has also been developed in consultation with the parent involvement panel and guided by the literature review (see online supplemental material 2: Interview topic guide). The interviews will aim to answer the following questions: (1) What are parents’ experiences following stillbirth? (2) What issues (outcomes) are important to parents after they have experienced a stillbirth? (3) What outcomes do parents think are important to measure so stillbirth care can be improved through research? Interviews will be audio and/or video recorded and transcribed verbatim. Stillbirth is a sensitive topic, and it is possible parents may experience distress during the interviews; should this happen, they will be offered the opportunity to pause the interview and, if they choose, to stop it completely. They will be signposted to support from their own healthcare provider or community support services.

10.1136/bmjopen-2021-056629.supp2 Supplementary data

Data collection and analysis will be guided by an iterative approach, allowing data analysis of early interviews to enrich data collection of later interviews. Following a familiarisation process, data will be coded blinded and in duplicate. Each line of the transcript will be coded systematically, identifying outcomes anchored in the words of the participant. Using an inductive approach, a codebook will be generated, and the data will be managed using NVivo software which will help to organise emergent themes. A constant comparative method will be adopted, whereby transcripts will be reread, and codes compared with every other occurrence in the interviews. Data will be analysed and conceptualised into broader categories using the ‘One sheet of paper’ technique^40^ and the DIPEx (personal experiences of health experiences and illness) techniques for coding.^40^ This approach has been taken to generate a deeper understanding and meaning of the outcomes, in the context of the lived experience of stillbirth, using the detail-rich interview transcripts. A collaborative approach will be taken with the analysis whereby emergent themes and codes will be developed iteratively with input from members of the project steering committee. The Consolidated criteria for Reporting Qualitative research checklist will be used to report the findings of the qualitative interviews.^41^

### Stage 2: creation of outcome long-list and pilot with think-aloud interviews

#### Creation of outcome long-list

A comprehensive outcome inventory will be developed from all the outcomes identified in the data extraction of the systematic review and analysis of the qualitative interviews. As an initial step, we will group similar definitions (extracting the wording description verbatim) under the same outcome name.^23^ Outcomes will then be grouped into outcome domains or categories to classify the broad aspects of the effects of interventions or care.^23^ The outcomes will be organised into outcome categories using an adapted taxonomy that has been developed for outcomes in medical research to help improve knowledge discovery.^42^ Each verbatim outcome definition will be categorised to an outcome name and mapped to a domain independently by two researchers from multi-professional backgrounds (a healthcare professional and a health service research methodologist) to provide transparency. Any differences will be resolved by consulting a senior member of the research team.

Consideration will be given to the order of questions and the number of items as previous research has demonstrated that question order could affect response rates and actual responses to question items.^43^ The final outcome long-list will be reviewed by the steering committee and parent involvement panel. Furthermore, with input from the parent involvement panel plain language definitions will be developed for each outcome item.

#### Pilot and think-aloud interviews

The questionnaire items and response scale format will be piloted using the think-aloud approach to ensure the ease of completion, readability, understandability and acceptability by stakeholders prior to recruitment.^23 44 45^ It will also be used to refine the long-list of outcomes. The think-aloud method has been used by other core outcome set developers to improve their questionnaire design.^45–49^ We will examine how parents and other stakeholders interpret the outcome labels and definitions, check they understand how to complete the nine-point Likert rating scale and identify problems.^23^ Participants will think aloud as they work through the draft Delphi and provide a running commentary on their thoughts on rating of outcomes.^23^ The interviewer will use open-ended cognitive probes as described in the interview guide (see online supplemental material 3: Think aloud topic guide). The probes will ascertain comprehension, retrieval, confidence judgement and responses to questions.^45^ We will also determine the length of time it takes to complete the survey to ensure response fatigue is minimal.

10.1136/bmjopen-2021-056629.supp3 Supplementary data

Interviews will be face to face or via Zoom teleconferencing and will be audio recorded once informed consent has been obtained. Transcribed interviews will be coded, by two independent researchers according to a framework of think-aloud categories.^50^ The coded comments will be subsequently tabulated in a ‘table of changes’ and for each outcome to provide a transparent method of recording suggestions (see online supplemental material 4: Table of changes for think-aloud interviews and questionnaire development). Suggested changes in wording, reasons for change and agreed changes will be documented providing transparency in the questionnaire development. This approach has been used in think-aloud interviews within the Person-Based Approach to intervention development.^51 52^ An iterative approach will be adopted; we will revise the questionnaire following analysis of an initial sample of think-aloud interviews, conduct further interviews, and revise the questionnaire until data saturation and no further changes are indicated. We estimate that we will interview approximately 12–15 stakeholders. Following these interviews, the final Delphi questionnaire will be produced.

10.1136/bmjopen-2021-056629.supp4 Supplementary data

### Stage 3: international Delphi survey

The core outcome set will be determined using a modified Delphi method. The Delphi methodology has been used to allow stakeholders with expert knowledge on a particular subject to achieve convergence of opinion on the importance of different outcomes using sequential questionnaires or face-to-face meetings.^23^ Responses for each outcome will be summarised and fed back anonymously in the following questionnaire round. Participants will be able to consider the responses of others and their previous response before rescoring each item; this has the benefit of allowing participants to review previous round results independently, with the overall aim to achieve consensus.

#### Selection and recruitment of stakeholders

Representatives from all stakeholder groups will be invited to participate in the think-aloud interviews, the Delphi survey and consensus meetings. Stakeholders will include two main groups: parents with a lived experience of stillbirth and professionals. The professional stakeholder group will include healthcare professionals caring for parents who have experienced stillbirth (eg, obstetricians, midwives, general practitioners, sonographers, psychiatrists, psychologists and doulas), researchers, bereavement charity representatives and stillbirth advocates. Due to translation costs and financial limitations of the study, non-English speakers will be excluded. A stakeholder recruitment sampling frame will be created to ensure there is maximum variation in the sample.

As stillbirth occurs globally, participants will be sought through an international network of parent support groups, organisations, professional associations and charities, including from high-income, low-income and middle-income countries. We will aim to achieve representation from most continents including Europe, Africa, Asia, Australia, North America and South America. We will aim to recruit a diverse range of mothers and fathers/partners who have experienced a stillbirth at a range of gestations and time periods since the stillbirth occurred. Family members of parents who experience stillbirth, for example grandparents, siblings or other immediate family member will also be eligible to participate. Parents will be identified via charity support groups, social media and the ISA. We will work with international collaborators in participating countries to use websites and social media that are most relevant to parents that we wish to approach. Healthcare professionals will be identified via email distribution lists using links with the Royal College of Obstetricians and Gynaecologists, the Royal College of Midwives, the ISA, the British Psychological Society (counselling, health psychology and clinical psychology divisions), Royal College of General Practitioners and British Association for Counselling and Psychotherapy. Researchers will be identified through authors of papers in the systematic review, and research networks.

#### Sample size

There are no generally accepted guidelines for the optimal size to achieve a consensus in Delphi Studies. Decisions about on how many individuals to include in a Delphi process is pragmatic, and not based on statistical power.^23 53^ Careful consideration will be made to sample stakeholders with a breadth of experience. For the Delphi survey, a minimum of 100 participants per stakeholder group (100 parents and 100 professionals) will be recruited to account for a 20% drop-out rate.^54 55^ This estimate is based on the typical response rate found from a review of published and ongoing studies that included Delphi to develop a core outcome set^54^ We will use evidence-based methods for maximising recruiting and retaining participants between rounds, for example, direct personalised email invitations, promotional animation and demonstration videos for each round of the Delphi and adopting a minimum waiting time between rounds 1 and 2.^54–56^

#### Delphi survey

Respondents will be invited to complete two sequential rounds of the Delphi survey via email. Study data will be collected and managed using REDCap (Research Electronic Data Capture) tools hosted at the University of Bristol.^57^ REDCap is a secure, web-based application designed to support data capture for research studies, providing: (1) an intuitive interface for validated data entry; (2) audit trails for tracking data manipulation and export procedures; (3) automated export procedures for seamless data downloads to common statistical packages and (4) procedures for importing data from external sources.^57^ Informed consent will be obtained via REDCap from all participants who agree to take part. The data will be analysed using SPSSS Version 28.0.^57^

Participants will be asked to indicate the importance of each outcome using a nine point Likert scale devised by the Grading of Recommendations Assessment Development and Evaluations working group.^58^ They will also be given the opportunity to add additional outcomes to be incorporated into round 2 of the survey. After round 1, data will be analysed using descriptive statistics to produce a summary of the results, including the presentation of the results in histograms. An anonymous summary of the responses will be fed back to participants according to each stakeholder group in round 2 of the survey and each participant will receive their own previous scores for round 1. Participants will be asked to reflect on the stakeholder group scores and their own score before rescoring each outcome and new outcomes identified by participants from round 1. Any outcomes not deemed important by the pre-specified criteria (see below) will be excluded. If a participant does not complete round 2 of the Delphi survey, their scores from round 1 will be counted as valid and retained in the study. The rate of missing responses will be reported with the results of the Delphi survey. The round 2 results will be reviewed by the steering committee to consider the need for a third Delphi survey round. Attrition bias will be assessed by comparing scores of those stakeholders completing both rounds of the Delphi survey, with those that only complete Round 1 alone. Scores will also be compared with those attending the consensus meetings compared with those not attending, to assess whether attendees of the consensus meeting are representative of those who participated in the survey.

#### Consensus definition

**Figure 3:**
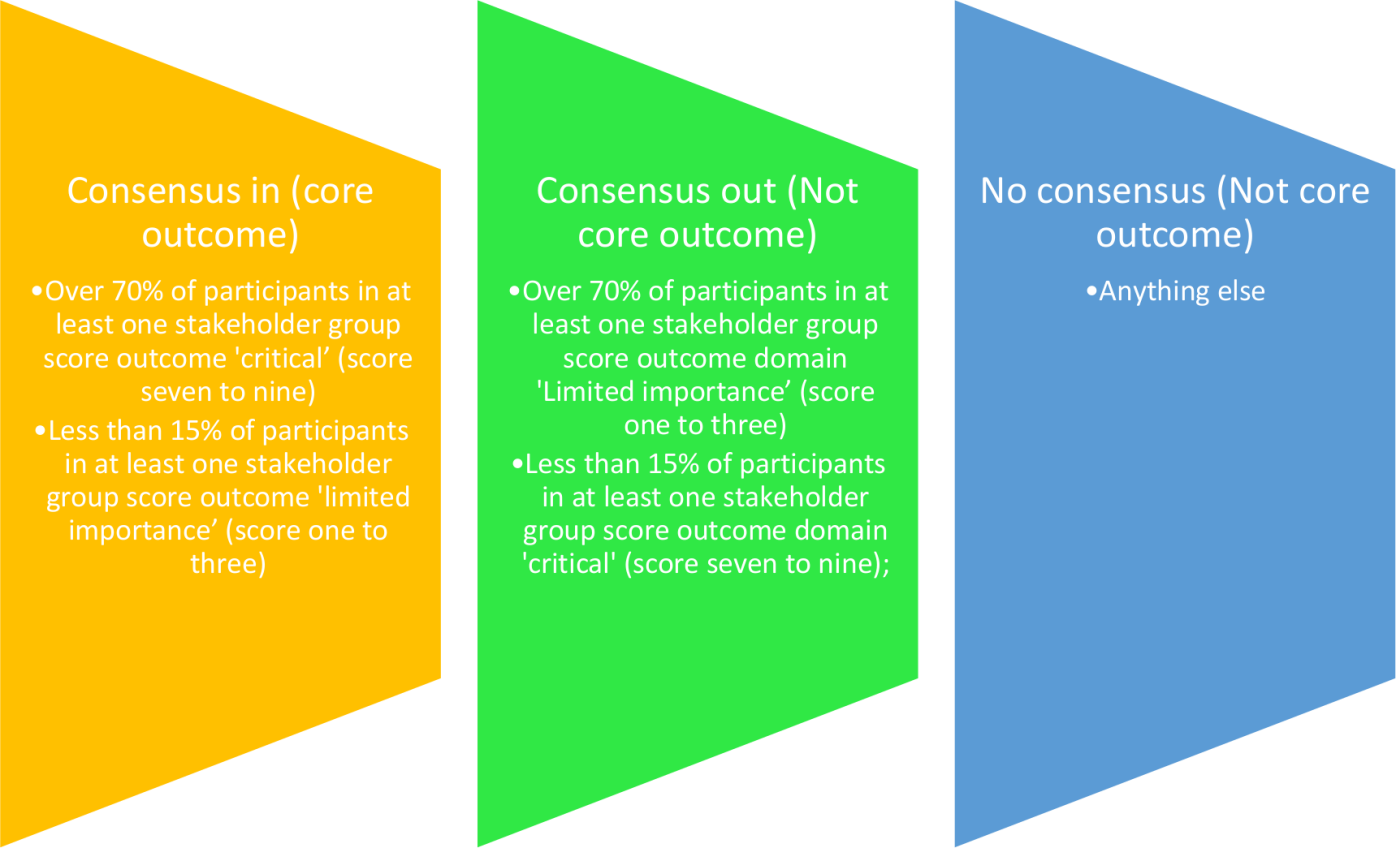
Consensus definition.

A standardised consensus definition will be applied to enable core outcomes to be identified: (1) ‘Consensus in’ (classify as a core outcome): Over 70% of participants in at least one stakeholder group score outcome 'critical’ (score seven to nine) and less than 15% of participants in at least one stakeholder group score outcome ‘limited importance’ (score one to three). (2) ‘Consensus out’ (do not classify as a core outcome): Over 70% of participants in at least one stakeholder group score outcome domain ‘limited importance’ (score 1–3) and less than 15% of participants in at least one stakeholder group score outcome domain ‘critical’ (score 7–9) or (3) ‘no Consensus’ (do not classify as a core outcome): anything else (see figure 3).^23 26^

The rationale for this definition is that for an outcome to be included in the core outcome set, it requires agreement by the majority that it is of critical importance and only a small minority consider it to have little importance. This definition will be reviewed by the steering committee after round 1 of the Delphi if a large proportion of outcomes are classified as ‘consensus in’. Possible strategies that could be adopted to be more stringent in the definition could include, having a higher percentage cut-off of stakeholders who need to score an outcome seven to nine to be ‘Consensus in’ (80% of participants in at least one stakeholder group) or deciding an outcome to be ‘critical’ only if scored eight to nine. Particular caution will be applied in the review of this definition to ensure that variation in parents’ views is not lost between rounds.

### Stage 4: consensus meetings to decide the core outcome set

At least two consensus meetings will take place to discuss the results of the survey and agree the final core outcome set. Stakeholders will be asked if they are willing to participate in the consensus meetings at the end of the Delphi questionnaire and will invited once the analysis of round 2 has been completed. If a large number of stakeholders are interested in attending the meetings, we will aim to have minimum representation from each continent and each stakeholder group. It is anticipated that these meetings will be either face to face or virtually via Zoom teleconferencing software and informed consent will be taken prior to commencement of each meeting. The meetings will be run sensitively by researchers and a bereavement care midwife who are experienced in running research meetings with bereaved parents.^9 59^ A representative from the Sands Charity and ISA will also be present for the meeting to support parents if required. The initial meeting will take place only with parents. This pre-meeting will allow parents to have the equal opportunity to voice their opinions without intimidation or influence from the other stakeholder groups. A subset of parent representatives will be invited to the second consensus meeting (and potentially third consensus meeting) with all stakeholder groups.

A modified nominal group technique will be used to further prioritise consensus outcomes.^60^ This technique ensures that all participants have the opportunity to provide their perspectives and to hear the views of others. The modified nominal group technique does not rely on statistical power. It is anticipated that 8–10 participants from each stakeholder group will participate in the consensus meetings, as this number has yielded sufficient results in the development of previous core outcome sets.^61 62^

Prior to the meeting attendees will be sent a reminder of their own personal Delphi score. A facilitator will present the results from the earlier rounds according to each stakeholder group. All potential core outcomes reaching the standardised definition for ‘Consensus in’ will be discussed. Participants in the meeting will be either asked to work individually or split into small groups or pairs to consider the outcomes, including any outcomes that they feel are missing. All the participants are then brought together to discuss each outcome in turn. Each participant will be asked to contribute their opinions on outcomes considered for inclusion in the final core outcome set. With consent of the participants the consensus meetings will be audio and video recorded and minuted.

A further round of voting and discussion will take place with the aim of achieving consensus and ratifying the final core outcome set. Items will be categorised as ‘Consensus in—outcome included in the final core outcome set’, ‘Consensus out—outcome not included in the final core outcome set’ or ‘No consensus—outcomes for which opinions on inclusion are divided’. This will be facilitated by online, smartphone or electronic keypad technology, allowing for all present to vote anonymously and simultaneously. Outcomes will be rejected where there is again ‘No consensus’ reached at this stage. The transcribed meeting will be uploaded onto NVivo and analysed using a content analysis to contextualise the decision making around the development of the core outcome set.^63^

### Identifying outcome measurement tools using the literature

Once the core outcome set is agreed it is important to determine how outcomes should be measured so that the core outcome set can be fully used.^21 23^ Currently, there are no guidelines available to support outcome measurement instrument selection for core outcome sets. Future research will include identifying potential outcome measurement tools for each outcome in the core outcome set from the systematic review. If no outcome measurement tools are identified for a core outcome using this method, this will be acknowledged, and identification and/or development, quality assessment and selection of suitable outcome measurement tools will form part of future research work.

## Ethics and dissemination

### Stage 5: share and promote: dissemination

We are aiming for this core outcome to be used in all future stillbirth care research. The dissemination strategy will be developed with the steering committee, the parent involvement panel and the University of Bristol’s Public Engagement Office. A range of methods will be used to raise awareness of the core outcome set and promote its adoption. The results of the systematic review, qualitative interviews, think-aloud interviews, the Delphi process and consensus meetings will be published in peer-reviewed specialty journals. An overview of the core outcome set will be disseminated to the CROWN and COMET initiatives. The results will be presented at national and international scientific conferences of the ISA, Royal College of Obstetricians and Gynaecologists, Royal College of Midwives, International Federation of Gynecology and Obstetrics, British Maternal and Fetal Society, the and the COMET conferences. Furthermore, we will promote a high-level awareness of the study and the core outcome set through social media via parent organisations and charities. Results will also be directly shared with professional associations, relevant university research departments and clinical guideline developers to maximise uptake of the final core outcome set.

#### Ethics

Ethical approval for the qualitative interviews has been approved by Berkshire Ethics Committee REC Referene 12/SC/0495.

Ethical approval for the think-aloud interviews, Delphi survey and consensus meetings has been awarded from the University of Bristol Faculty of Health Sciences Research Ethics Committee (Reference number: 116535).

## Supplementary Material

Reviewer comments

Author's manuscript
