# Supplementary material for: Protocol for the development of a core outcome set for stillbirth care research (iCHOOSE Study)
Source: BMJ Open. 2022 Feb 9;12(2):e056629. doi: 10.1136/bmjopen-2021-056629 (PMC8830254; doi:10.1136/bmjopen-2021-056629)
Supplement: Supplementary data [file bmjopen-2021-056629supp001.pdf]

Core Outcome Set-STAndardised Protocol Items (COS-STAP) Checklist for the iCHOOSE Study

Item Number	Name	Location
1	Identify in the title that the paper describes the protocol for the planned development of a COS	TITLE/ABSTRACT<br>Page 1
2	Provide a structured abstract	ABSTRACT<br>Page 1-2
3	Describe the background and explain the rationale for developing the COS	INTRODUCTION<br>Page 3-4
P	Describe the specific objectives with reference to developing a COS	INTRODUCTION and AIMS AND OBJECTIVES<br>Page 4
5	Describe the health condition(s) and population(s) that will be covered by the COS	INTRODUCTION AND METHODS AND ANALYSIS<br>Page 5<br><br>[Scope – Health Condition and Population]
6	Describe the intervention(s) that will be covered by the COS	INTRODUCTION AND METHODS AND ANALYSIS<br>[Scope – Intervention]<br>Page 5
7	Describe the setting(s) that will be covered by the COS	INTRODUCTION AND METHODS AND ANALYSIS<br>Page 5
8	Indicate the COS study registration details and registry name. If not yet registered indicate the intended registry	REGISTRATION DETAILS<br>Page 2
9	Describe any study oversight committees	STEERING COMMITTEE AND PATIENT AND PUBLIC INVOLVEMENT<br>Page 5 & 6
10	Describe sources of funding, role of funders	FUNDING STATEMENT Page 13-14
11	Describe any potential conflicts of interest within the study team and how these will be managed	CONFLICTS OF INTEREST<br>Page 14
12	Describe the stakeholder groups to be involved in the COS development process and the rationale for their involvement	METHODS –STAKEHOLDERS<br>Page 9-10

13	Describe the eligibility criteria for individuals from each stakeholder group	METHODS –STAKEHOLDERS Page 9-10
14	Describe how individuals of each stakeholder groups will be identified	METHODS –STAKEHOLDERS Page 9-10
15	Describe how individuals of each stakeholder group will be chosen from within the stakeholder group	METHODS –STAKEHOLDERS Page 9-10
16	Describe how many planned individuals within each stakeholder group will be invited to participate in the consensus process	METHODS –STAKEHOLDERS [Participants – Sample Size] Page 10
17	Describe how individuals will be invited to take part in the consensus process	METHODS –STAKEHOLDERS Page 10
18	Describe the information sources that will be used to identify the list of outcomes. Outline the methods or reference other protocols/papers.	METHODS – SYSTEMATIC REVIEW AND QUALITATIVE INTERVIEWS Page 6&7
19	Describe how outcomes may be dropped/combined, with reasons	METHODS Page 11
20	Describe the methods to identify outcome descriptor terms	METHODS Page 8&9
21	Describe the plans for how the consensus process will be undertaken	METHODS Page 9-12
22	Describe what information will be presented to participants at the start of the consensus process	METHODS Page 8-9
23	Describe what each participant will be asked to do at each stage of the consensus process	METHODS Page 11
24	Describe how the participants will receive any feedback during the consensus process	METHODS Page 11
25	Describe how non-response (or partial response) will be handled during the consensus process	METHODS Page 11
26	Describe how the study material will be made patient friendly and understandable (if relevant)	METHODS Page 8-9
27	Describe the consensus definition	METHODS Page 11-12
28	Describe the procedure for determining how outcomes will be added/combined/dropped from consideration during the consensus process	METHODS Page 11
29	Describe how outcomes will be scored and summarised	METHODS Page 11

30	Describe how the response rate will be maximised	METHODS<br>Page 10
31	Describe how attrition bias will be assessed	METHODS<br>Page 11
32	Describe any software that will be used during the consensus process and to analyse the results	METHODS<br>Page 10
33	Describe any plans for obtaining research ethics committee / institutional review board approval in relation to the consensus process (if relevant)	ETHICS/ DISSEMINATION<br>Page 13
34	Describe how informed consent will be obtained (if relevant)	ETHICS/ DISSEMINATION & METHODS<br>Page 7,9, 10, 11,12
35	Describe any details about how the confidentiality of data collection will be preserved during the consensus process (if relevant)	METHODS<br>Page 10-11
