# Supplementary material for: Protocol for the development of a core outcome set for stillbirth care research (iCHOOSE Study)
Source: BMJ Open. 2022 Feb 9;12(2):e056629. doi: 10.1136/bmjopen-2021-056629 (PMC8830254; doi:10.1136/bmjopen-2021-056629)
Supplement: Supplementary data [file bmjopen-2021-056629supp003.pdf]

Supplementary material 3: Think aloud topic guide

Interview Guide

Harmonising outcomes for research and care after stillbirth (The iCHOOSE Study)

Think-aloud interviews for the development of a core outcome set questionnaire

Introduction to study

These interviews aim to find out about how you go about completing a questionnaire to develop a core outcome set. You will be asked to provide a running commentary on how you rate the importance of individual outcomes. We are particularly interested in how you understand the questions, how difficult or easy it is to rate the outcomes, the wording of the outcomes and any changes you would make to the questionnaire. We are also interested in whether there are additional outcomes that should be included in the questionnaire.

If at any time you don't want to continue the interview, you are free to tell me that you wish to stop, and we can either take a break or you can stop completely. It's fine for you to do that.

Interview Instructions

Participants will be read the following instructions, adapted from Green and Gilhooly (1996) and French et al. (2007):

We are interested in how people complete the following questionnaire. This questionnaire will ask you to rank outcomes on how important they are to you to measure in research and evaluating care after stillbirth. We want to check that people understand the questions in the way that we meant them. To do this, I am going to ask you to 'think aloud' as you complete the questionnaire. What I mean by 'think aloud' is that I want you to tell me everything you are thinking as you read each question and decide how to answer it. I would like you to talk aloud constantly. I don't want you to plan out what you say or try to explain to me what you are saying. Just act as if you are alone in the room speaking to yourself. If you are silent for any long period of time, I will ask you to talk or ask you question to help you. Please try to speak as clearly as possible, as I shall be recording you as you speak. Do you understand what I want you to do?

Interview question probes (Adapted from Collins (2003), French et al (2007) and McCorry (2013))

General

- Tell me what are you thinking?
- How did you go about rating that outcome?
- How easy or difficult did you find this outcome to rate?
- Would you like to make any changes to this question/outcome?

Comprehension

- What does that outcome mean to you?
- What did you understand by this outcome?
- Are there any problems with the wording of this outcome?

Retrieval

- How did you calculate your answer?
- Is this outcome relevant to a particular time period that you can relate to?
- How applicable is this outcome to your individual circumstances?

Confidence judgement

- How sure of your answer are you?

Response

- How did you feel about answering this question?

Additional outcomes

- Would you like to include any other additional outcomes?
