# Supplementary material for: Protocol for the development of a core outcome set for stillbirth care research (iCHOOSE Study)
Source: BMJ Open. 2022 Feb 9;12(2):e056629. doi: 10.1136/bmjopen-2021-056629 (PMC8830254; doi:10.1136/bmjopen-2021-056629)
Supplement: Supplementary data [file bmjopen-2021-056629supp004.pdf]

Supplementary material 4: Table of changes for think aloud interviews and questionnaire development

Delphi Questionnaire for core outcome set for research after stillbirth					
Negative Comments	*Positive Comments*	*Possible Change*	*Reason for change*	*Agreed change*	*MoScoW*
