# Supplementary material for: Protocol for the development of a core outcome set for stillbirth care research (iCHOOSE Study)
Source: BMJ Open. 2022 Feb 9;12(2):e056629. doi: 10.1136/bmjopen-2021-056629 (PMC8830254; doi:10.1136/bmjopen-2021-056629)
Supplement: Author's manuscript [file bmjopen-2021-056629.draft_revisions.pdf]

BMJ Open is committed to open peer review. As part of this commitment we make the peer review history of every article we publish publicly available.

When an article is published we post the peer reviewers' comments and the authors' responses online. We also post the versions of the paper that were used during peer review. These are the versions that the peer review comments apply to.

The versions of the paper that follow are the versions that were submitted during the peer review process. They are not the versions of record or the final published versions. They should not be cited or distributed as the published version of this manuscript.

BMJ Open is an open access journal and the full, final, typeset and author-corrected version of record of the manuscript is available on our site with no access controls, subscription charges or pay-per-view fees (http://bmjopen.bmj.com).

If you have any questions on BMJ Open's open peer review process please email info.bmjopen@bmj.com

BMJ Open

Protocol for the development of a core outcome set for stillbirth care research (iCHOOSE Study)

Journal:	*BMJ Open*
Manuscript ID	bmjopen-2021-056629
Article Type:	Protocol
Date Submitted by the Author:	24-Aug-2021
Complete List of Authors:	Bakhbakhi, Danya; University of Bristol Medical School, Translational Health Sciences<br>Fraser, Abigail; University of Bristol Medical School, Population Health Sciences<br>Siasakos, Dimitris; UCL, UCL EGA Institute for Women's Health<br>Hinton, Lisa; THIS Institute<br>Davies, Anna; University of Bristol Medical School, Translational Health Sciences<br>Merriel, Abi; University of Bristol Medical School, Population Health Sciences<br>Duffy, James; King's Fertility, The Fetal Medicine Research Institute<br>Redshaw, Maggie; NPEU, Department of Population Health<br>Lynch, Mary ; University of Bristol Medical School<br>Timlin, Laura; North Bristol NHS Trust, Women & Children's Health<br>Flenady, Vicki; Mater Research Institute-University of Queensland,<br>Heazell, Alexander; University of Manchester, Maternal and Fetal Health Research Centre<br>Downe, Soo; University of Central Lancashire, research in childbirth and health<br>Slade, Pauline; University of Liverpool, Psychological Sciences<br>Brookes, Sara; University of Birmingham<br>Wojcieszek, Aleena; Mater Research Institute - The University of Queensland, Centre of Research Excellence in Stillbirth<br>Murphy, Margaret; University College Cork National University of Ireland, Nursing and Midwifery<br>de Oliveira Salgado, Heloisa; University of São Paulo<br>Pollock, Danielle; The University of Adelaide, Public Health<br>Aggarwal, Neelam ; Post Graduate Institute of Medical Education and Research<br>Attachie, Irene; University of Health and Allied Sciences School of Public Health<br>Leisher, Susannah; International Stillbirth Alliance, ;<br>Kihusa, Wanijiru; Still a mum<br>Mulley , Kate ; Sands<br>Wimmer, Lindsey ; Star Legacy Foundation<br>., UK iCHOOSE parent involvement group<br>Burden, Christy; University of Bristol Medical School, Translational Health Sciences
Keywords:	OBSTETRICS, QUALITATIVE RESEARCH, Quality in health care < HEALTH SERVICES ADMINISTRATION & MANAGEMENT, Protocols & guidelines <

	HEALTH SERVICES ADMINISTRATION & MANAGEMENT, Fetal medicine < OBSTETRICS, Maternal medicine < OBSTETRICS

For peer review only - http://bmjopen.bmj.com/site/about/guidelines.xhtml

Title

Protocol for the development of a core outcome set for stillbirth care research (iCHOOSE Study)

Danya Bakhbakhi, University of Bristol, danya.bakhbakhi@bristol.ac.uk
Abigail Fraser, University of Bristol, abigail.fraser@bristol.ac.uk
Dimitris Siasakos, University College London, d.siasakos@ucl.ac.uk
Lisa Hinton, University of Cambridge, lisa.hinton@thisinstitute.cam.ac.uk
Anna Davies, University of Bristol, anna.davies@bristol.ac.uk
Abi Merriel, University of Bristol, abi.merriel@bristol.ac.uk
James M N Duffy, King's Fertility, james.duffy3@nhs.net
Maggie Redshaw, University of Oxford, maggie.redshaw@npeu.ox.ac.uk
Mary Lynch, University of Bristol, mary.lynch@nbt.nhs.uk
Laura Timlin, North Bristol NHS Trust, laura.timlin@nbt.nhs.uk
Vicki Flenady, The University of Queensland, vicki.flenady@mater.uq.edu.au
Alexander Heazell, University of Manchester, alexander.heazell@manchester.ac.uk
Soo Downe, University of Central Lancashire, sdowne@uclan.ac.uk
Pauline Slade, University of Liverpool, pauline.slade@liverpool.ac.uk
Sara Brookes, University of Birmingham, s.t.brookes@bham.ac.uk
Aleena Wojcieszek, The University of Queensland, aleena.wojcieszek@uqconnect.edu.au
Margaret Murphy, University College Cork, mgt.murphy@ucc.ie
Heloisa de Oliveira Salgado, University of Sao Paulo, hellosalgado@gmail.com
Danielle Pollock, JBI, University of Adelaide, danielle.pollock@adelaide.edu.au
Neelam Aggarwal, Post Graduate Institute of Medical Education and Research, Chandigarh, India, drneelamaggarwal@gmail.com
Irene Attachie, University of Health and Allied Sciences, Ghana, iattachie@uhas.edu.gh
Susannah Hopkins Leisher, International Stillbirth Alliance, shleisher@aol.com
Wanjiru Kihusa, Still a Mum, Kenya, wanjiru@stillamum.com
Kate Mulley, Sands Charity, United Kingdom, kate.mulley@sands.org.uk
Lindsey Wimmer, Star Legacy Foundation, lindsey@starlegacyfoundation.org
UK iCHOOSE parent involvement group

Christy Burden, University of Bristol, christy.burden@bristol.ac.uk

Corresponding Author: Dr Danya Bakhbakhi, danya.bakhbakhi@bristol.ac.uk, Centre for Academic Women's Health, University of Bristol, Chilterns, Women's Health, Southmead Hospital, Westbury on Trym, Bristol BS10 5NB, United Kingdom, 0117 950 5050

Keywords

Stillbirth, Consensus, Pregnancy, Research design, Core outcome set

Word count

4929

Abstract

For peer review only - http://bmjopen.bmj.com/site/about/guidelines.xhtml

Introduction

Stillbirth is associated with significant physical, psychosocial and economic consequences for parents, families, wider society and the healthcare system. There is emerging momentum to design and evaluate interventions for care after stillbirth and in subsequent pregnancies. However, there is insufficient evidence to inform clinical practice compounded by inconsistent outcome reporting in research studies. To address this paucity of evidence, we plan to develop a core outcome set for stillbirth care research, through an international consensus process with key stakeholders including parents, healthcare professionals and researchers.

Methods and analysis

The development of this core outcome set will be divided into five distinct phases: 1) Identifying potential outcomes from a mixed-methods systematic review and analysis of interviews with parents who have experienced stillbirth; 2) Creating a comprehensive outcome long-list and piloting of a Delphi questionnaire using think-aloud interviews; 3) Choosing the most important outcomes by conducting an international two-round Delphi survey including high-, middle- and low- income countries; 4) Deciding the core outcome set by consensus meetings with key stakeholders; and 5) Dissemination and promotion of the core outcome set. A parent and public involvement panel and international steering committee has been convened to co-produce every stage of the development of this core outcome set.

Ethics and dissemination

Ethical approval for the qualitative interviews has been approved by Berkshire Ethics Committee REC Reference 12/SC/0495. Ethical approval for the think-aloud interviews, Delphi survey and consensus meetings has been awarded from the University of Bristol Faculty of Health Sciences Research Ethics Committee (Reference number:116535). The dissemination strategy is being developed with the parent and public involvement panel and steering committee. Results will be published in peer-reviewed speciality journals, shared at national and international conferences and promoted through parent organisations and charities.

Registration details

COMET Initiative registration number 775
PROSPERO registration number CRD42018087748

Article summary

STRENGTHS AND LIMITATIONS OF THIS STUDY

- Using robust and transparent methodology, this will be the first core outcome set developed for use in stillbirth care research, which will ultimately improve evidence synthesis in this field and could reduce research wastage.

For peer review only - http://bmjopen.bmj.com/site/about/guidelines.xhtml

- Identification of outcomes reported in experimental, observational and qualitative studies will ensure all published outcomes within the literature are considered for inclusion in the core outcome set. Furthermore, in-depth qualitative interviews with parents will enable the identification of novel and parent-important outcomes not identified from the systematic review.
- Parent representation is a strength of this study. We are including bereaved parent stakeholders at every stage of the development, co-producing the research with a parent involvement panel, and with parent representation within the project steering committee.
- Qualitative interviews (in Stage 1) include UK parents only. However, we are triangulating these with outcomes identified in the systematic review of global literature and think aloud interviews are not limited to parents in the UK. Furthermore, we have international parent representation on the steering committee to help mitigate this limitation and increase the generalisability of the results.
- International stakeholders will be recruited for the Delphi survey and consensus meetings. However, due to funding limitations and translation costs, the survey and consensus meetings will be conducted in the English language only. Future research will endeavour to validate the core outcome set in languages other than English.

Introduction

Worldwide it is estimated that there are 2 million stillbirths every year[1]. Stillbirth is associated with significant physical, psychosocial, health and economic costs for parents, their families, wider society and the healthcare system[2,3,4]. In a subsequent pregnancy, a history of stillbirth has been shown to be associated with higher frequencies of adverse clinical outcomes, including increased risk of stillbirth recurrence, antenatal complications, mental health concerns and impact on subsequent children[5,6,7,8.] The negative consequences of stillbirth are widespread and long-lasting; therefore, it is important to invest in high-quality research to enable healthcare professionals and researchers to deliver the best care for affected families.

Several care-related interventions are available to minimise the negative impact of stillbirth. These interventions can be implemented from the immediate identification of a stillbirth to when parents are discharge from hospital to the community or in a subsequent pregnancy. Examples include, supporting parents' choices around birth and afterwards, offering opportunities for parents to make memories with their baby, support with post-mortem investigation decision making, engagement of the parents in the perinatal mortality review process[9–12], bereavement care from healthcare professionals[13], counselling and specialist care in subsequent pregnancies[14]. Yet very little is known about the effectiveness of these interventions [15].

There is momentum to research, design and evaluate interventions to improve care for parents following stillbirth and in any subsequent pregnancies[15,16,17.] However, systematic reviews suggest few methodologically rigorous studies exist to inform clinical practice and their results cannot be synthesised quantitatively due to a high degree of heterogeneity of outcome reporting[15,17,18]. In 2018, a Cochrane review on care prior to and during subsequent pregnancies following stillbirth for improving outcomes, found insufficient and inconsistent evidence to inform clinical practice[17]. The authors of this review concluded that it is important to have

For peer review only - http://bmjopen.bmj.com/site/about/guidelines.xhtml
For peer review only

consistency in data collection across all future trials and this may be facilitated by a core outcome set for stillbirth care research[17].

A core outcome set is a consensus-derived minimum set of outcomes that should be measured and reported in all research studies of a specific disease or trial population[19]. It does not preclude the measurement of additional specific outcomes; however, a minimum set of outcomes will allow higher quality of evidence to identify the most effective interventions and care packages offered. A recent web-based survey of healthcare professionals, researchers and advocates identified the development of a core outcomes set for stillbirth (and recurrent stillbirth) research as one of the top five priority research topics to inform clinical practice for the care of families following stillbirth[20]. Currently there are no available core outcome sets published for stillbirth care research (i.e. research focusing on care after a stillbirth is identified) https://www.comet-initiative.org/Studies.

The inclusion of patients in the development of a core outcome set is paramount as they are the key stakeholders in the research outcomes. Inclusion of parents can lead to a widening of the research agenda, identifying important patient reported outcomes and recognising previously neglected patient outcomes that matter to those who experience stillbirth[21]. There is a need to develop and evaluate evidence-based interventions using outcomes that directly relate to bereaved parents' experiences. To enable this, it is essential to establish a minimum set of outcomes that includes parents and relevant stakeholders in the development process. If applied in clinical trials, a core outcome set for stillbirth care research developed with stakeholder input, will provide a tool to give consistency in outcome measurement, minimise reporting bias, and allow for direct comparison of interventions and care across research studies. This could lead to better evidence being produced to improve clinical decision making in the future.

Aim and objectives

AIM

The iCHOOSE study aims to develop a minimum set of outcomes that should be evaluated and reported in all future stillbirth care research in high-, middle- and low- income country settings, through an international consensus process of key stakeholders including parents, healthcare professionals, researchers and charity representatives.

OBJECTIVES

1. To investigate what outcomes are reported in existing studies assessing the impact of stillbirth on parents.
2. To investigate parental experiences following stillbirth and identify important outcomes for bereaved parents not reported in the scientific literature.
3. To pilot and develop a Delphi questionnaire, using think-aloud interviews.
4. To achieve international consensus on a core outcome set for stillbirth care research using a Delphi survey technique and stakeholder consensus meetings.
5. To disseminate and promote the core outcome set for stillbirth care research.

For peer review only - http://bmjopen.bmj.com/site/about/guidelines.xhtml

Methods and analysis

There is no standardised way to develop a core outcome set[21]. The COMET initiative has collated methodological resources to assist with the development of the core outcome set including a systematic review outlining the issues to consider[21–24]. COMET resources, including the COMET Handbook: version 1.0 and reviewed published core outcome sets have been used to inform the study design[23–30]. This study is prospectively registered on the COMET website https://www.comet-initiative.org/studies/details/775. The Core Outcome Set-STAndards for Development (COS-STAD) and the Core Outcome Set-STAndardised Protocol Items (COS-STAP) have been followed in the planning of the methods of this core outcome set project[31,32]. **See Supplementary material 1: Core Outcome Set-STAndardised Protocol Items (COS-STAP) Checklist for the iCHOOSE Study.** The final core outcome set will be reported in accordance with the Core Outcome Set-STAndards for Reporting statement (COS-STAR)[33].

SCOPE OF THIS CORE OUTCOME SET

Health condition and population

The core outcome set will be applicable to families who have experienced a stillbirth in a singleton or multiple pregnancy. We will aim for this core outcome set to be applicable to all countries internationally including high-, middle- and low- income countries. The definition of stillbirth varies internationally and therefore the gestation will be dependent on the study setting. It is our intention that this core outcome set could be applied to stillbirths from at least 20 weeks' gestation, including antepartum and intrapartum stillbirths from any cause including due to a congenital abnormality. We will set exclude outcomes related to the termination of pregnancy and neonatal death population.

Interventions

The core outcome set will be relevant to all stillbirth care research. Stillbirth care research includes the care that parents (and families) receive after a stillbirth has been identified. The core outcome set will not be limited by the type of intervention or the setting in which it is delivered. It will cover all medical and psychosocial interventions and care parents are offered following a stillbirth and in a subsequent pregnancy[15,17]. **See Figure 1: Types of interventions after stillbirth that should be evaluated using outcomes identified in the core outcome set.**

Context

The core outcome set will be developed for use in all stillbirth care research (e.g.randomised controlled trials, observational studies and systematic reviews). It is also anticipated that it could be utilised in the evaluation of clinical practice guidelines, care pathways for bereaved parents and training for healthcare professional[34].

PATIENT AND PUBLIC INVOLVEMENT

For peer review only - http://bmjopen.bmj.com/site/about/guidelines.xhtml

Parent perspectives are integral to every stage of the development, including the input into this protocol, the systematic review, qualitative interviews, Delphi survey, consensus meeting and dissemination of results. A parent involvement panel has been established and training is being provided using methods exemplified by NIHR INVOLVE. The parent involvement panel have also co-designed the parent animation video to aid recruitment https://vimeo.com/292143259/f2edb109dd.

STEERING COMMITTEE

An international expert steering committee including healthcare professionals, parents with a lived experience of stillbirth, charity representatives and researchers with diverse expertise has been convened to guide the research design, recruitment and development of the core outcome set. This group has stakeholder representation from Europe, Australia, North America, South America, Africa and Asia.

COLLABORATIONS

We have established the International Collaboration for Harmonising Outcomes fOr Stillbirth research and carE (iCHOOSE) initiative. The iCHOOSE collaboration aims to develop a core outcome set for stillbirth care research with the overall aim of improving outcomes for parents and the wider family. This collaboration is endorsed by the Core Outcomes in Women's Health (CROWN) initiative; the Medical Sociology and Health Experiences Research Group, University of Oxford; the National Stillbirth Centre for Research Excellence, Australia, The Stillbirth and Neonatal Death Charity (Sands); Tommy's National Centre for Maternity Improvement, Twins Trust, Star Legacy Foundation and International Stillbirth Alliance.

STUDY OVERVIEW

The study will be divided into five distinct stages. **See Figure 2: iCHOOSE Study overview**

STAGE 1: IDENTIFYING POTENTIAL OUTCOMES

Systematic review: What outcomes have been reported?

Previously reported outcomes and associated outcome measurement tools relevant to stillbirth care research are being identified through a systematic review of the literature. Our systematic review process has been prospectively registered on PROSPERO International prospective register of systematic reviews (CRD42018087748). The electronic databases MEDLINE, PubMed, Embase, Scopus, Amed, BNI, CINAHL, PsycINFO Cochrane Register of Controlled Trials will be searched from 1998 to present**.** Reference lists of extracted articles will also be searched. We will include all randomised trials, observational and qualitative studies that report an outcome following stillbirth. Case reports, editorials, review articles, abstracts and grey literature will be excluded. Studies including mothers, fathers, children, siblings, and grandparents experiencing a stillbirth in a singleton or multiple pregnancy will be included. Studies will not be excluded based on the gestational definition of stillbirth, as the definition varies between jurisdictions. Titles, abstracts and full texts

For peer review only - http://bmjopen.bmj.com/site/about/guidelines.xhtml

of studies will be screened independently by two review authors using Covidence systematic review software[35]. Disagreements will be resolved through a third reviewer.

A standardised, pre-piloted electronic data extraction form has been developed to extract data. Data will be extracted in duplicate and includes basic publication details (including author and date of publication); study setting; study population; details of intervention (if applicable); study methodology; outcomes measured verbatim, their definition (if stated), their relevant outcome measurement tool (if applicable) and whether the tool is validated for that cultural context and if parents and members of the public were involved in the outcome selection. A sequential explanatory approach will be undertaken i.e., outcomes from quantitative studies will be extracted initially followed by outcomes reported in the qualitative literature. This will be done to compare, and contrast outcomes reported in the qualitative literature. A comprehensive inventory of outcomes reported will be developed from the data extraction The systematic review will be reported using the Preferred Reporting Items for Systematic Reviews and Meta-Analyses (PRISMA) guidelines[36].

Qualitative interviews: What outcomes are important to parents?

Capturing patient perspectives is crucial in the development of a core outcome set as they often identify outcomes not considered by other stakeholders or within the literature [37]. Parents with a lived experience of stillbirth in the United Kingdom (UK) will be recruited to participate in qualitative interviews through Sands, National Health Service Hospital Trusts, the Twins Trust, bereavement support groups, the parent involvement panel and snowballing through personal contacts of the research team and the parent involvement panel. To ensure diverse opinions participants will be purposively sampled for maximum variation. Participants will include mothers and fathers/partners from a wide range of social, ethnic, and cultural backgrounds who have experienced a stillbirth at a range of gestations and time periods since the stillbirth occurred. Parents who have a personal history of a stillbirth at more than 24 weeks' gestation (UK definition), at least six months prior to the study would be eligible to participate. This definition was chosen as recent research has focused on parents' experiences of care following the death of a baby in pregnancy between 20 and 24 weeks in the UK[38]. The findings of this research will be incorporated into the systematic review findings. Furthermore, as we are only recruiting UK parents, we plan to triangulate the data with outcomes extracted from the qualitative data from the systematic review. Parents will be interviewed individually or jointly, according to preference. The number of parents recruited will depend on when theoretical saturation is reached (i.e. when no new themes emerge) [39].

With informed consent, semi-structured interviews with parents will be conducted in either parents' homes, a suitable private location of their choice or via Zoom teleconference software. A researcher with training in qualitative interview methods will conduct the interviews (DB) supported by an experienced qualitative researcher (LH). The interviews will invite parents to narrate their lived experienced of stillbirth. However, an interview topic guide has also been developed in consultation with the parent involvement panel and guided by the literature review **(See Supplementary material 2: Interview topic guide).** The interviews will aim to answer the following questions: 1) What are parents' experiences following stillbirth? 2) What issues (outcomes) are important to parents after they have experienced a stillbirth? 3) What outcomes do parents think are important to measure so stillbirth care can be improved through research? Interviews will be audio and/or video recorded

For peer review only - http://bmjopen.bmj.com/site/about/guidelines.xhtml

and transcribed verbatim. Stillbirth is a sensitive topic, and it is possible parents may experience distress during the interviews; should this happen, they will be offered the opportunity to pause the interview and, if they choose, to stop it completely. They will be signposted to support from their own healthcare provider or community support services.

Data collection and analysis will be guided by an iterative approach, allowing data analysis of early interviews to enrich data collection of later interviews. Following a familiarisation process, data will be coded blinded and in duplicate. Each line of the transcript will be coded systematically, identifying outcomes anchored in the words of the participant. Using an inductive approach, a codebook will be generated, and the data will be managed using NVivo software which will help to organise emergent themes. A constant comparative method will be adopted, whereby transcripts will be re-read, and codes compared with every other occurrence in the interviews. Data will be analysed and conceptualised into broader categories using the 'One sheet of paper' technique[40] and the DIPEx (personal experiences of health experiences and illness) techniques for coding[40]. This approach has been taken to generate a deeper understanding and meaning of the outcomes, in the context of the lived experience of stillbirth, using the detail-rich interview transcripts. A collaborative approach will be taken with the analysis whereby emergent themes and codes will be developed iteratively with input from members of the project steering committee. The Consolidated criteria for reporting qualitative research (COREQ) checklist will be used to report the findings of the qualitative interviews[41].

STAGE 2: CREATION OF OUTCOME LONG-LIST AND PILOT WITH THINK ALOUD INTERVIEWS

Creation of outcome long-list

A comprehensive outcome inventory will be developed from all the outcomes identified in the data extraction of the systematic review and analysis of the qualitative interviews. As an initial step, we will group similar definitions (extracting the wording description verbatim) under the same outcome name[23]. Outcomes will then be grouped into outcome domains or categories to classify the broad aspects of the effects of interventions or care[23]. The outcomes will be organised into outcome categories using an adapted taxonomy that has been developed for outcomes in medical research to help improve knowledge discovery[42]. Each verbatim outcome definition will be categorised to an outcome name and mapped to a domain independently by two researchers from multi-professional backgrounds (a health care professional and a health service research methodologist) to provide transparency. Any differences will be resolved by consulting a senior member of the research team.

Consideration will be given to the order of questions and the number of items as previous research has demonstrated that question order could affect response rates and actual responses to question items[43]. The final outcome long-list will be reviewed by the steering committee and parent involvement panel. Furthermore, with input from the parent involvement panel plain language definitions will be developed for each outcome item.

Pilot and think-aloud interviews

For peer review only - http://bmjopen.bmj.com/site/about/guidelines.xhtml

The questionnaire items and response scale format will be piloted using the think-aloud approach to ensure the ease of completion, readability, understandability and acceptability by stakeholders prior to recruitment[23,44,45]. It will also be used to refine the long-list of outcomes. The think-aloud method has been used by other core outcome set developers to improve their questionnaire design[45–48,49]. We will examine how parents and other stakeholders interpret the outcome labels and definitions, check they understand how to complete the nine-point Likert rating scale and identify problems[23]. Participants will think aloud as they work through the draft Delphi and provide a running commentary on their thoughts on rating of outcomes[23]. The interviewer will use open-ended cognitive probes as described in the interview guide **(See Supplementary material 3: Think aloud topic guide).** The probes will ascertain comprehension, retrieval, confidence judgement and responses to questions[45]. We will also determine the length of time it takes to complete the survey to ensure response fatigue is minimal.

Interviews will be face-to-face or via Zoom teleconferencing and will be audio recorded once informed consent has been obtained. Transcribed interviews will be coded, by two independent researchers according to a framework of think-aloud categories[50]. The coded comments will be subsequently tabulated in a 'table of changes' and for each outcome to provide a transparent method of recording suggestions **(See Supplementary material 4: Table of changes for think aloud interviews and questionnaire development).** Suggested changes in wording, reasons for change and agreed changes will be documented providing transparency in the questionnaire development. This approach has been used in think-aloud interviews within the Person-Based Approach to intervention development[51,52]. An iterative approach will be adopted; we will revise the questionnaire following analysis of an initial sample of think aloud interviews, conduct further interviews, and revise the questionnaire until data saturation and no further changes are indicated. We estimate that we will interview approximately 12 to 15 stakeholders. Following these interviews, the final Delphi questionnaire will be produced.

STAGE 3: INTERNATIONAL DELPHI SURVEY

The core outcome set will be determined using a modified Delphi method. The Delphi methodology has been used to allow stakeholders with expert knowledge on a particular subject to achieve convergence of opinion on the importance of different outcomes using sequential questionnaires or face-to-face meetings[23]. Responses for each outcome will be summarised and fed back anonymously in the following questionnaire round. Participants will be able to consider the responses of others and their previous response before re-scoring each item; this has the benefit of allowing participants to review previous round results independently, with the overall aim to achieve consensus.

Selection and recruitment of stakeholders

Representatives from all stakeholder groups will be invited to participate in the think-aloud interviews, the Delphi survey and consensus meetings. Stakeholders will include two main groups: parents with a lived experience of stillbirth and professionals. The professional stakeholder group will include healthcare professionals caring for parents who have experienced stillbirth (e.g. obstetricians, midwives, general practitioners, sonographers, psychiatrists, psychologists and doulas), researchers, bereavement charity

For peer review only - http://bmjopen.bmj.com/site/about/guidelines.xhtml

representatives and stillbirth advocates. Due to translation costs and financial limitations of the study, non-English speakers will be excluded. A stakeholder recruitment sampling frame will be created to ensure there is maximum variation in the sample.

As stillbirth occurs globally, participants will be sought through an international network of parent support groups, organisations, professional associations and charities, including from high, low- and middle- income countries. We will aim to achieve representation from most continents including Europe, Africa, Asia, Australia, North America and South America. We will aim to recruit a diverse range of mothers and fathers/partners who have experienced a stillbirth at a range of gestations and time periods since the stillbirth occurred. Family members of parents who experience stillbirth, for example grandparents, siblings or other immediate family member will also be eligible to participate. Parents will be identified via charity support groups, social media, and the International Stillbirth Alliance. We will work with international collaborators in participating countries to use websites and social media that are most relevant to parents that we wish to approach. Healthcare professionals will be identified via email distribution lists using links with the Royal College of Obstetricians and Gynaecologists, the Royal College of Midwives, the International Stillbirth Alliance, the British Psychological Society (counselling, health psychology and clinical psychology divisions), Royal College of General Practitioners and British Association for Counselling and Psychotherapy. Researchers will be identified through authors of papers in the systematic review, and research networks.

Sample size

There are no generally accepted guidelines for the optimal size to achieve a consensus in Delphi Studies. Decisions about on how many individuals to include in a Delphi process is pragmatic, and not based on statistical power[23,53]. Careful consideration will be made to sample stakeholders with a breadth of experience. For the Delphi survey, a minimum of 100 participants per stakeholder group (100 parents and 100 professionals) will be recruited to account for a 20% drop-out rate[54,55]. This estimate is based on the typical response rate found from a review of published and ongoing studies that included Delphi to develop a core outcome set[54] We will use evidence-based methods for maximizing recruiting and retaining participants between rounds, for example, direct personalised email invitations, promotional animation and demonstration videos for each round of the Delphi and adopting a minimum waiting time between rounds one and two[54–56].

Delphi Survey

Respondents will be invited to complete two sequential rounds of the Delphi survey via email. Study data will be collected and managed using REDCap (Research Electronic Data Capture) tools hosted at the University of Bristol[57]. REDCap is a secure, web-based application designed to support data capture for research studies, providing: 1) an intuitive interface for validated data entry; 2) audit trails for tracking data manipulation and export procedures; 3) automated export procedures for seamless data downloads to common statistical packages; and 4) procedures for importing data from external sources[57]. Informed consent will be obtained via REDCap from all participants who agree to take part. The data will be analysed using STATA[57].

Participants will be asked to indicate the importance of each outcome using a nine point Likert scale devised by the Grading of Recommendations Assessment Development and Evaluations (GRADE) working group [(42)]. They will also be given the opportunity to add additional outcomes to be incorporated into round two of the survey.

For peer review only - http://bmjopen.bmj.com/site/about/guidelines.xhtml

After round one, data will be analysed using descriptive statistics to produce a summary of the results, including the presentation of the results in histograms. An anonymous summary of the responses will be fed back to participants according to each stakeholder group in round two of the survey and each participant will receive their own previous scores for round one. Participants will be asked to reflect on the stakeholder group scores and their own score before rescoring each outcome and new outcomes identified by participants from round one. Any outcomes not deemed important by the pre-specified criteria (see below) will be excluded. If a participant does not complete round two of the Delphi survey, their scores from round one will be counted as valid and retained in the study. The rate of missing responses will be reported with the results of the Delphi survey. The round two results will be reviewed by the steering committee to consider the need for a third Delphi survey round.

Consensus definition

A standardised consensus definition will be applied to enable core outcomes to be identified: [1] 'Consensus in' (classify as a core outcome): Over 70% of participants in at least one stakeholder group score outcome 'critical' (score seven to nine) and less than 15% of participants in at least one stakeholder group score outcome 'limited importance' (score one to three). [2] 'Consensus out' (do not classify as a core outcome): Over 70% of participants in at least one stakeholder group score outcome domain 'limited importance' (score one to three) and less than 15% of participants in at least one stakeholder group score outcome domain 'critical' (score seven to nine); or [3] 'No Consensus' (do not classify as a core outcome): Anything else[23,26]. **See figure 3: Consensus definition.**

The rationale for this definition is that for an outcome to be included in the core outcome set, it requires agreement by the majority that it is of critical importance and only a small minority consider it to have little importance. This definition will be reviewed by the steering Committee after Round 1 of the Delphi if a large proportion of outcomes are classified as 'Consensus in'. Possible strategies that could be adopted to be more stringent in the definition could include, having a higher percentage cut-off of stakeholders who need to score an outcome seven to nine to be 'Consensus in' (80% of participants in at least one stakeholder group) or deciding an outcome to be 'critical' only if scored eight to nine. Particular caution will be applied in the review of this definition to ensure that variation in parents' views is not lost between rounds.

STAGE 4: CONSENSUS MEETINGS TO DECIDE THE CORE OUTCOME SET

At least two consensus meetings will take place to discuss the results of the survey and agree the final core outcome set. Stakeholders will be asked if they are willing to participate in the consensus meetings at the end of the Delphi questionnaire and will invited once the analysis of round two has been completed. If a large number of stakeholders are interested in attending the meetings, we will aim to have minimum representation from each continent and each stakeholder group. It is anticipated that these meetings will be either face-to-face or virtually via Zoom teleconferencing software and informed consent will be taken prior to commencement of each meeting. The meetings will be run sensitively by researchers and a bereavement care midwife who are experienced in running research meetings with bereaved parents[9,59]. A representative from the Sands Charity and International Stillbirth Alliance will also be present for the meeting to support parents if

For peer review only - http://bmjopen.bmj.com/site/about/guidelines.xhtml

required. The initial meeting will take place only with parents. This pre-meeting will allow parents to have the equal opportunity to voice their opinions without intimidation or influence from the other stakeholder groups. A sub-set of parent representatives will be invited to the second consensus meeting (and potentially third consensus meeting) with all stakeholder groups.

A modified Nominal Group Technique will be used to further prioritise consensus outcomes[60]. This technique ensures that all participants have the opportunity to provide their perspectives and to hear the views of others. The modified Nominal Group Technique does not rely on statistical power. It is anticipated that eight to ten participants from each stakeholder group will participate in the consensus meetings, as this number has yielded sufficient results in the development of previous core outcome sets[61],[62].

Prior to the meeting attendees will be sent a reminder of their own personal Delphi score. A facilitator will present the results from the earlier rounds according to each stakeholder group. All potential core outcomes reaching the standardised definition for 'Consensus in' will be discussed. Participants in the meeting will be either asked to work individually or split into small groups or pairs to consider the outcomes, including any outcomes that they feel are missing. All the participants are then brought together to discuss each outcome in turn. Each participant will be asked to contribute their opinions on outcomes considered for inclusion in the final core outcome set. With consent of the participants the consensus meetings will be audio and video recorded and minuted.

A further round of voting and discussion will take place with the aim of achieving consensus and ratifying the final core outcome set. Items will be categorised as 'Consensus in – outcome included in the final core outcome set', 'Consensus out – outcome not included in the final core outcome set' or 'No consensus - outcomes for which opinions on inclusion are divided'. This will be facilitated by online, smartphone or electronic keypad technology, allowing for all present to vote anonymously and simultaneously. Outcomes will be rejected where there is again 'No consensus' reached at this stage. The transcribed meeting will be uploaded onto NVivo and analysed using a content analysis to contextualise the decision making around the development of the core outcome set[63].

STAGE 5: SHARE AND PROMOTE

Dissemination

We are aiming for this core outcome to be used in all future stillbirth care research. The dissemination strategy will be developed with the steering committee, the parent involvement panel, and the University of Bristol's Public Engagement Office. A range of methods will be used to raise awareness of the core outcome set and promote its adoption. The results of the systematic review, qualitative interviews, think-aloud interviews, the Delphi process and consensus meetings will be published in peer-reviewed speciality journals. An overview of the core outcome set will be disseminated to the Core Outcomes in Women's Health (CROWN) and Core Outcome Measures in Effectiveness Trials (COMET) initiatives. The results will be presented at national and international scientific conferences of the International Stillbirth Alliance (ISA), Royal College of Obstetricians and Gynaecologists, Royal College of Midwives, International Federation of Gynecology and Obstetrics, British

For peer review only - http://bmjopen.bmj.com/site/about/guidelines.xhtml

Maternal & Fetal Society, the and the COMET conferences. Furthermore, we will promote a high-level awareness of the study and the core outcome set through social media via parent organisations and charities. Results will also be directly shared with professional associations, relevant university research departments and clinical guideline developers to maximise uptake of the final core outcome set.

Identifying outcome measurement tools using the literature

Once the core outcome set is agreed it is important to determine how outcomes should be measured so that the core outcome set can be fully utilised[21,23]. Currently there are no guidelines available to support outcome measurement instrument selection for core outcome sets. Future research will include identifying potential outcome measurement tools for each outcome in the core outcome set from the systematic review. If no outcome measurement tools are identified for a core outcome using this method, this will be acknowledged, and identification and/or development, quality assessment and selection of suitable outcome measurement tools will form part of future research work.

Ethics

Ethical approval for the qualitative interviews has been approved by Berkshire Ethics Committee REC Referene 12/SC/0495.

Ethical approval for the think-aloud interviews, Delphi survey and consensus meetings has been awarded from the University of Bristol Faculty of Health Sciences Research Ethics Committee (Reference number:116535).

AUTHOR CONTRIBUTIONS

Study concept: DB, CB, AF, AD, DS, LH, JMND, SB: Study Design: DB, CB, AF, AD, DS, LH, JMND, SB MR, AH, VF, ML, AM, LT, SD, PS, AW, MM, HOS, DP, NA, SL, IA, WK, KM, LKD, LT, LW, UKPIG. Drafting of the article: DB. Critical revision of the article for important intellectual content: DB, CB, AF, AD, DS, LH, JMND, SB MR, AH, VF, ML, AM, LT SD, PS, AW, MM, HOS, DP, NA, SL, IA, WK, KM, LKD, LT, LW, UKPIG. Study supervision: CB, AF, DS, LH, AD

ACKNOWLEDGEMENTS
The authors thank the UK iCHOOSE parent involvement panel for their involvement in the co-production of this research project: Lisa Thorne, Will Hatton, Carla Mereu Keating, Heather Jane Coombs, Dave Coombs, Michelle Fischer, Ali Fischer, Fraser Morton, Naomi Hepworth.

FUNDING STATEMENT

This report is independent research arising from a doctoral fellowship (DRF) supported by the National Institute for Health Research (NIHR DRF-2017-10-130). The views expressed in this publication are those of the authors and not necessarily those of the National Health Service, the National Institute for Health Research, or the Department of Health.

For peer review only - http://bmjopen.bmj.com/site/about/guidelines.xhtml

DATA STATEMENT

None available as protocol only.

COMPETING INTERESTS STATEMENT

Dr Aleena Wojcieszek has received consulting fees from the Sillbirth CRE. There are no other competing interests to declare.

References

1. Unicef. Unicef Stillbirth data. 2020.
2. Heazell AEP, Siassakos D, Blencowe H, Burden C, Bhutta ZA, Cacciatore J. Stillbirths: economic and psychosocial consequences. Lancet [internet]. Elsevier. 2017;387.
3. Burden C, Bradley S, Storey C, Ellis A, Heazell AEP, Downe S, et al. From grief, guilt pain and stigma to hope and pride -- a systematic review and meta-analysis of mixed-method research of the psychosocial impact of stillbirth. BMC Pregnancy Childbirth [Internet]. 2016;16(1):9. Available from: http://dx.doi.org/10.1186/s12884-016-0800-8
4. Sarkar A, Siwatch S, Aggarwal N, Singla R, Grover S. The unheard parental cry of a stillbirth: fathers and mothers. Arch Gynecol Obstet [Internet]. 2021; Available from: https://doi.org/10.1007/s00404-021-06120-9
5. Black M, Shetty A, Bhattacharya S. Obstetric outcomes subsequent to intrauterine death in the first pregnancy. Bjog [Internet]. 2008;115. Available from: http://dx.doi.org/10.1111/j.1471-0528.2007.01562.x
6. Lamont K, Scott NW, Jones GT, Bhattacharya S. Risk of recurrent stillbirth: systematic review and meta-analysis. BMJ (Clinical Res Ed [Internet]. 2015;350(8014):h3080. Available from: http://www.hlisd.org/LibraryDetail.aspx?LibraryID=3872
7. Hughes P, Turton P, Hopper E, McGauley GA, Fonagy P. Disorganised attachment behaviour among infants born subsequent to stillbirth. J Child Psychol Psychiatry [Internet]. 2001;42(6):791–801. Available from: http://www.hlisd.org/LibraryDetail.aspx?LibraryID=3872
8. Mills TA, Ricklesford C, Cooke A, Heazell AEP, Whitworth M, Lavender T. Parents' experiences and expectations of care in pregnancy after stillbirth or neonatal death: a metasynthesis. BJOG [Internet]. 2014;121. Available from: http://dx.doi.org/10.1111/1471-0528.12656
9. Burden C, Bakhbakhi D, Heazell AE, Lynch M, Timlin L, Bevan C, et al. Parents' Active Role and ENgagement in The review of their Stillbirth/perinatal death 2 (PARENTS 2) study: a mixed-methods study of implementation. BMJ Open [Internet]. 2021 Mar 1;11(3):e044563. Available from: http://bmjopen.bmj.com/content/11/3/e044563.abstract
10. Bakhbakhi D, Siassakos D, Storey C, Heazell A, Lynch M, Timlin L, et al. PARENTS 2 study protocol: pilot of Parents{\textquoteright} Active Role and ENgagement in the review of Their Stillbirth/perinatal death. BMJ Open [Internet]. 2018;8(1). Available from: https://bmjopen.bmj.com/content/8/1/e020164
11. Bakhbakhi D, Burden C, Storey C, Heazell AE, Lynch M, Timlin L, et al. PARENTS 2 Study: a qualitative study of the views of healthcare professionals and stakeholders on parental engagement in the perinatal mortality review{\textemdash}from {\textquoteleft}bottom of the pile{\textquoteright} to joint learning. BMJ Open [Internet]. 2018;8(11). Available from: https://bmjopen.bmj.com/content/8/11/e023792
12. Bakhbakhi D, Siassakos D, Burden C, Jones F, Yoward F, Redshaw M, et al. Learning from deaths:

For peer review only - http://bmjopen.bmj.com/site/about/guidelines.xhtml

Parents' Active Role and ENgagement in The review of their Stillbirth/perinatal death (the PARENTS 1 study). BMC Pregnancy Childbirth. 2017 Oct;17(1):333.
13. Siassakos D, Jackson S, Gleeson K, Chebsey C, Ellis A, Storey C. All bereaved parents are entitled to good care after stillbirth: a mixed-methods multicentre study (INSIGHT). BJOG [Internet]. 2018;125(2):160. Available from: http://ovidsp.ovid.com/ovidweb.cgi?T=JS&PAGE=fulltext&MODE=ovid&CSC=Y&NEWS=N&D=ovft&SEARCH=1470-0328.is+and+%22125%22.vo+and+%222%22.ip+and+%22160%22.pg
14. Ladhani NNN, Fockler ME, Stephens L, Barrett JFR, Heazell AEP. No. 369-Management of Pregnancy Subsequent to Stillbirth. J Obstet Gynaecol Canada [Internet]. 2018 Dec 1;40(12):1669–83. Available from: https://doi.org/10.1016/j.jogc.2018.07.002
15. Koopmans L, Wilson T, Cacciatore J, Flenady V. Support for mothers, fathers and families after perinatal death. Cochrane Database Syst Rev [Internet]. 2013;(6). Available from: http://dx.doi.org/10.1002/14651858.CD000452.pub3
16. Gynaecologists. RC of O and. Each Baby Counts: 2015 Summary Report.
17. Wojcieszek AM, Shepherd E, Middleton P, Lassi ZS, Wilson T, Murphy MM, et al. Care prior to and during subsequent pregnancies following stillbirth for improving outcomes. Cochrane Database Syst Rev [Internet]. 2018;(12). Available from: https://doi.org//10.1002/14651858.CD012203.pub2
18. Hennegan JM, Henderson J, Redshaw M. Contact with the baby following stillbirth and parental mental health and well-being: a systematic review. BMJ Open [Internet]. 2015 Nov 1;5(11). Available from: http://bmjopen.bmj.com/content/5/11/e008616.abstract
19. Clarke M. Standardising outcomes for clinical trials and systematic reviews. Trials [Internet]. 2007;8(1):39. Available from: http://dx.doi.org/10.1186/1745-6215-8-39
20. Wojcieszek AM, Heazell AEP, Middleton P, Ellwood D, Silver RM, Flenady V. Research priorities and potential methodologies to inform care in subsequent pregnancies following stillbirth: a web-based survey of healthcare professionals, researchers and advocates. BMJ Open [Internet]. 2019 Jun 1;9(6):e028735. Available from: http://bmjopen.bmj.com/content/9/6/e028735.abstract
21. Williamson PR, Altman DG, Blazeby JM, Clarke M, Devane D, Gargon E, et al. Developing core outcome sets for clinical trials: issues to consider. Trials [Internet]. 2012;13(1):132. Available from: http://dx.doi.org/10.1186/1745-6215-13-132
22. Initiative C. COMET [Internet]. [cited 2017 Jan 9]. Available from: http://www.comet-initiative.org/
23. Williamson PR, Altman DG, Bagley H, Barnes KL, Blazeby JM, Brookes ST, et al. The COMET Handbook: version 1.0. Trials [Internet]. 2017 Jun;18(3):280. Available from: https://doi.org/10.1186/s13063-017-1978-4
24. Gorst SL, Gargon E, Clarke M, Blazeby JM, Altman DG, Williamson PR. Choosing Important Health Outcomes for Comparative Effectiveness Research: An Updated Review and User Survey. PLoS One [Internet]. 2016 Jan 19;11(1):e0146444. Available from: http://dx.doi.org/10.1371%2Fjournal.pone.0146444
25. Boers M, Kirwan JR, Wells G, Beaton D, Gossec L, d'Agostino M-A, et al. Developing Core Outcome Measurement Sets for Clinical Trials: OMERACT Filter 2.0. J Clin Epidemiol [Internet]. 2017 Jan 9;67(7):745–53. Available from: http://dx.doi.org/10.1016/j.jclinepi.2013.11.013
26. Duffy JMN, van 't Hooft J, Gale C, Brown M, Grobman W, Fitzpatrick R, et al. A protocol for developing, disseminating, and implementing a core outcome set for pre-eclampsia. Pregnancy Hypertens An Int J Women's Cardiovasc Heal [Internet]. 2016;6(4):274–8. Available from: http://www.sciencedirect.com/science/article/pii/S2210778916300149
27. Potter S, Davies C, Holcombe C, Weiler-Mithoff E, Skillman J, Vidya R, et al. International development and implementation of a core measurement set for research and audit studies in implant-based breast reconstruction: a study protocol. BMJ Open [Internet]. 2020 Jan 1;10(1):e035505. Available from: http://bmjopen.bmj.com/content/10/1/e035505.abstract

For peer review only - http://bmjopen.bmj.com/site/about/guidelines.xhtml

28. Khalil A, Perry H, Duffy J, Reed K, Baschat A, Deprest J, et al. Twin–Twin Transfusion Syndrome: study protocol for developing, disseminating, and implementing a core outcome set. Trials [Internet]. 2017;18(1):325. Available from: https://doi.org/10.1186/s13063-017-2042-0
29. Webbe J, Brunton G, Ali S, Duffy JM, Modi N, Gale C. Developing, implementing and disseminating a core outcome set for neonatal medicine. BMJ Paediatr open [Internet]. 2017 Jul 26;1(1):e000048–e000048. Available from: https://pubmed.ncbi.nlm.nih.gov/29637104
30. Hirsch M, Duffy JMN, Kusznir JO, Davis CJ, Plana MN, Khan KS, et al. Variation in outcome reporting in endometriosis trials: a systematic review. Am J Obstet Gynecol [Internet]. 2016 Apr 1;214(4):452–64. Available from: https://doi.org/10.1016/j.ajog.2015.12.039
31. Kirkham JJ, Davis K, Altman DG, Blazeby JM, Clarke M, Tunis S, et al. Core Outcome Set-STAndards for Development: The COS-STAD recommendations. PLOS Med [Internet]. 2017 Nov 16;14(11):e1002447. Available from: https://doi.org/10.1371/journal.pmed.1002447
32. Kirkham JJ, Gorst S, Altman DG, Blazeby JM, Clarke M, Tunis S, et al. Core Outcome Set-STAndardised Protocol Items: the COS-STAP Statement. Trials [Internet]. 2019;20(1):116. Available from: https://doi.org/10.1186/s13063-019-3230-x
33. Kirkham JJ, Gorst S, Altman DG, Blazeby JM, Clarke M, Devane D, et al. Core Outcome Set–STAndards for Reporting: The COS-STAR Statement. PLOS Med [Internet]. 2016 Oct 18;13(10):e1002148. Available from: http://dx.doi.org/10.1371%2Fjournal.pmed.1002148
34. Dodd S, Harman N, Taske N, Minchin M, Tan T, Williamson PR. Core outcome sets through the healthcare ecosystem: the case of type 2 diabetes mellitus. Trials [Internet]. 2020;21(1):570. Available from: https://doi.org/10.1186/s13063-020-04403-1
35. Innovation VH. Covidence systematic review software. Melbourne, Australia;
36. Satement P. Preferred Reporting Items for Systematic Reviews and Meta-Analyses (PRISMA).
37. de Wit M, Abma T, Koelewijn-van Loon M, Collins S, Kirwan J. Involving patient research partners has a significant impact on outcomes research: a responsive evaluation of the international OMERACT conferences. BMJ Open [Internet]. 2013 Jan 1;3(5). Available from: http://bmjopen.bmj.com/content/3/5/e002241.abstract
38. Smith LK, Dickens J, Bender Atik R, Bevan C, Fisher J, Hinton L. Parents' experiences of care following the loss of a baby at the margins between miscarriage, stillbirth and neonatal death: a UK qualitative study. BJOG An Int J Obstet Gynaecol [Internet]. 2020 Jun 1;127(7):868–74. Available from: https://doi.org/10.1111/1471-0528.16113
39. Baker, S and Edwards R. How many qualitative interviews is enough? [Internet]. National Centre for Research Methods. [cited 2017 Jan 15]. Available from: http://eprints.ncrm.ac.uk/2273/4/how_many_interviews.pdf
40. Ziebland S, McPherson A. Making sense of qualitative data analysis: an introduction with illustrations from DIPEx (personal experiences of health and illness). Med Educ [Internet]. 2006 May 1;40(5):405–14. Available from: http://dx.doi.org/10.1111/j.1365-2929.2006.02467.x
41. Tong A, Sainsbury P, Craig J. Consolidated criteria for reporting qualitative research (COREQ): a 32-item checklist for interviews and focus groups. Int J Qual Heal Care [Internet]. 2007 Nov 19;19(6):349 LP – 357. Available from: http://intqhc.oxfordjournals.org/content/19/6/349.abstract
42. Dodd S, Clarke M, Becker L, Mavergames C, Fish R, Williamson PR. A taxonomy has been developed for outcomes in medical research to help improve knowledge discovery. J Clin Epidemiol [Internet]. 2017/12/28. 2018 Apr;96:84–92. Available from: https://pubmed.ncbi.nlm.nih.gov/29288712
43. Brookes ST, Chalmers KA, Avery KNL, Coulman K, Blazeby JM, Barham CP, et al. Impact of question order on prioritisation of outcomes in the development of a core outcome set: a randomised controlled trial. Trials [Internet]. 2018;19(1):66. Available from: https://doi.org/10.1186/s13063-017-2405-6
44. Drennan J. Cognitive interviewing: verbal data in the design and pretesting of questionnaires. J Adv Nurs [Internet]. 2003 Apr 1;42(1):57–63. Available from: https://doi.org/10.1046/j.1365-2648.2003.02579.x

For peer review only - http://bmjopen.bmj.com/site/about/guidelines.xhtml

45. Collins D. Pretesting survey instruments: An overview of cognitive methods. Qual Life Res [Internet]. 2003;12(3):229–38. Available from: https://doi.org/10.1023/A:1023254226592
46. Mathioudakis AG, Abroug F, Agusti A, Bakke P, Bartziokas K, Beghe B, et al. Core outcome set for the management of acute exacerbations of chronic obstructive pulmonary disease: the COS-AECOPD ERS Task Force study protocol. ERJ Open Res [Internet]. 2020 Jul 1;6(3):193–2020. Available from: http://openres.ersjournals.com/content/6/3/00193-2020.abstract
47. Srikandarajah N, Noble A, Clark S, Wilby M, Freeman BJC, Fehlings MG, et al. Cauda Equina Syndrome Core Outcome Set (CESCOS): An international patient and healthcare professional consensus for research studies. PLoS One [Internet]. 2020 Jan 10;15(1):e0225907–e0225907. Available from: https://pubmed.ncbi.nlm.nih.gov/31923259
48. Hagen NA, Stiles C, Nekolaichuk C, Biondo P, Carlson LE, Fisher K, et al. The Alberta Breakthrough Pain Assessment Tool for Cancer Patients: A Validation Study Using a Delphi Process and Patient Think-Aloud Interviews. J Pain Symptom Manage [Internet]. 2008;35(2):136–52. Available from: http://www.sciencedirect.com/science/article/pii/S088539240700677X
49. Willis GB. Cognitive interviewing: a tool for improving questionnaire design [Internet]. Thousand Oaks: Sage; 2005. Available from: https://doi.org/10.4135/9781412983655
50. French DP, Cooke R, Mclean N, Williams M, Sutton S. What Do People Think about When They Answer Theory of Planned Behaviour Questionnaires?: A `Think Aloud' Study. J Health Psychol [Internet]. 2007 Jul 1;12(4):672–87. Available from: https://doi.org/10.1177/1359105307078174
51. Bradbury K, Morton K, Band R, van Woezik A, Grist R, McManus RJ, et al. Using the Person-Based Approach to optimise a digital intervention for the management of hypertension. PLoS One [Internet]. 2018 May 3;13(5):e0196868. Available from: https://doi.org/10.1371/journal.pone.0196868
52. Yardley L, Ainsworth B, Arden-Close E, Muller I. The person-based approach to enhancing the acceptability and feasibility of interventions. Pilot Feasibility Stud [Internet]. 2015;1(1):37. Available from: https://doi.org/10.1186/s40814-015-0033-z
53. Calvert M, Blazeby J, DG A, al et. Reporting of patient-reported outcomes in randomized trials: The consort pro extension. JAMA [Internet]. 2013 Feb 27;309(8):814–22. Available from: http://dx.doi.org/10.1001/jama.2013.879
54. Gargon E, Crew R, Burnside G, Williamson PR. Higher number of items associated with significantly lower response rates in COS Delphi surveys. J Clin Epidemiol [Internet]. 2019;108:110–20. Available from: http://www.sciencedirect.com/science/article/pii/S0895435618307765
55. Hall DA, Smith H, Heffernan E, Fackrell K, Group for the COM in TID (COMiT'ID) RS. Recruiting and retaining participants in e-Delphi surveys for core outcome set development: Evaluating the COMiT'ID study. PLoS One [Internet]. 2018 Jul 30;13(7):e0201378. Available from: https://doi.org/10.1371/journal.pone.0201378
56. Alkhaffaf B, Blazeby JM, Metryka A, Glenny A-M, Adeyeye A, Costa PM, et al. Methods for conducting international Delphi surveys to optimise global participation in core outcome set development: a case study in gastric cancer informed by a comprehensive literature review. Trials [Internet]. 2021;22(1):410. Available from: https://doi.org/10.1186/s13063-021-05338-x
57. Harris PA, Taylor R, Thielke R, Payne J, Gonzalez N, Conde JG. Research electronic data capture (REDCap)—A metadata-driven methodology and workflow process for providing translational research informatics support. J Biomed Inform [Internet]. 2009;42(2):377–81. Available from: http://www.sciencedirect.com/science/article/pii/S1532046408001226
58. Group GW. GRADE [Internet]. [cited 2017 Jan 9]. Available from: http://www.gradeworkinggroup.org
59. Bakhbakhi D, Siassakos D, Lynch M, Timlin L, Storey C, Heazell A, et al. PARENTS 2 Study: consensus report for parental engagement in the perinatal mortality review process. Ultrasound Obstet Gynecol [Internet]. 2018 Oct 7;0(ja). Available from: https://doi.org/10.1002/uog.20139
60. Harvey N, Holmes CA. Nominal group technique: An effective method for obtaining group consensus.

For peer review only - http://bmjopen.bmj.com/site/about/guidelines.xhtml

Int J Nurs Pract [Internet]. 2012 Apr 1;18(2):188–94. Available from: https://doi.org/10.1111/j.1440-172X.2012.02017.x
61. Perry H, Duffy JMN, Umadia O, Khalil A. Outcome reporting across randomized trials and observational studies evaluating treatments for twin–twin transfusion syndrome: systematic review. Ultrasound Obstet Gynecol [Internet]. 2018;52(5):577–85. Available from: https://obgyn.onlinelibrary.wiley.com/doi/abs/10.1002/uog.19068
62. Duffy JMN, AlAhwany H, Bhattacharya S, Collura B, Curtis C, Evers JLH, et al. Developing a core outcome set for future infertility research: an international consensus development study†‡. Hum Reprod [Internet]. 2020 Dec 1;35(12):2725–34. Available from: https://doi.org/10.1093/humrep/deaa241
63. Hsieh H-F, Shannon SE. Three Approaches to Qualitative Content Analysis. Qual Health Res [Internet]. 2005 Nov 1;15(9):1277–88. Available from: https://doi.org/10.1177/1049732305276687

For peer review only

For peer review only - http://bmjopen.bmj.com/site/about/guidelines.xhtml

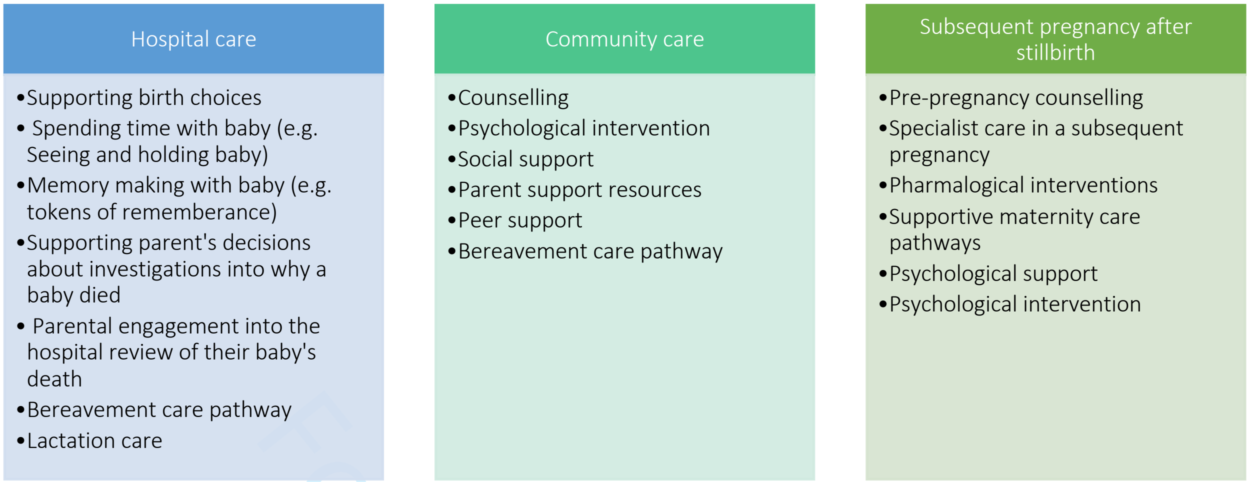

Figure 1: Types of interventions after stillbirth that should be evaluated using outcomes identified in the core outcome set

For peer review only

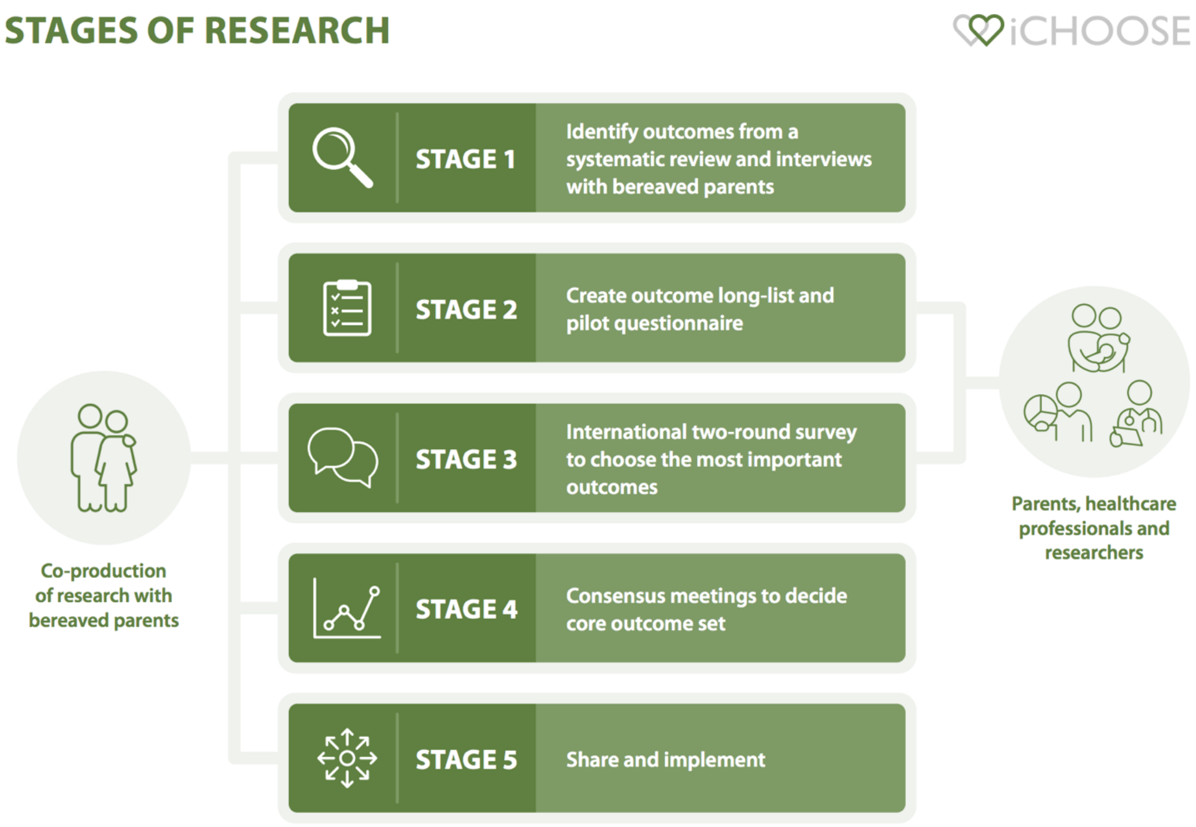

Figure 2: iCHOOSE Study overview

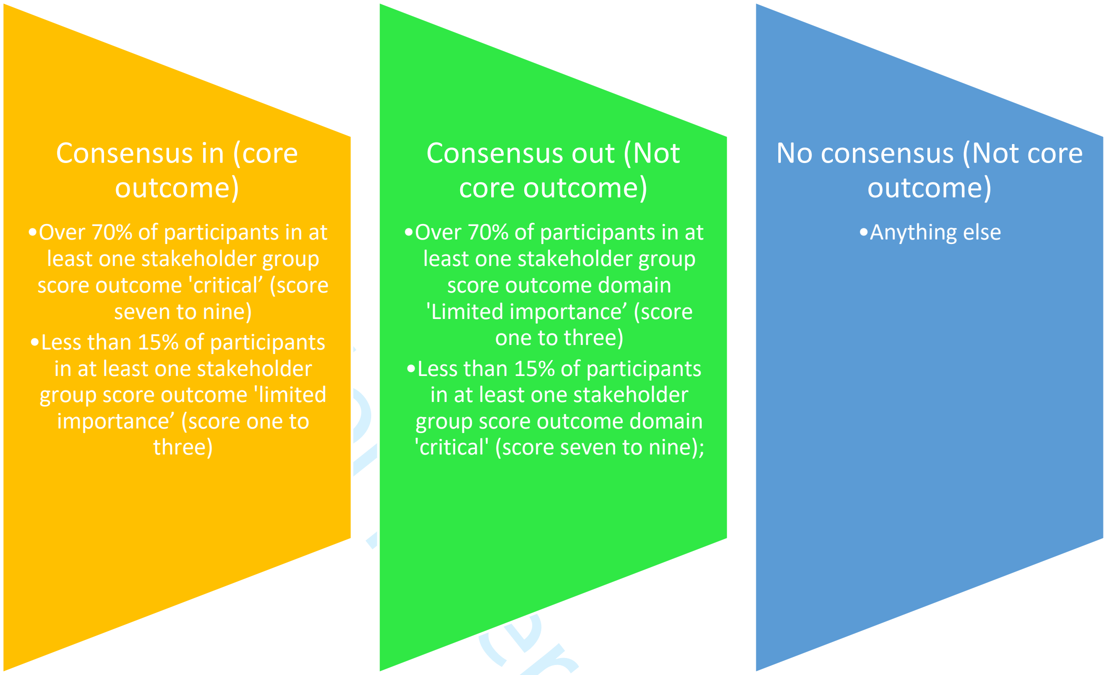

Figure 3: Consensus Definition

For peer review only

Core Outcome Set-STAndardised Protocol Items (COS-STAP) Checklist for the iCHOOSE Study

Item Number	Name	Location
1	Identify in the title that the paper describes the protocol for the planned development of a COS	TITLE/ABSTRACT
2	Provide a structured abstract	ABSTRACT
3	Describe the background and explain the rationale for developing the COS	INTRODUCTION
4	Describe the specific objectives with reference to developing a COS	INTRODUCTION and AIMS AND OBJECTIVES
5	Describe the health condition(s) and population(s) that will be covered by the COS	INTRODUCTION AND METHODS AND ANALYSIS<br><br>[Scope – Health Condition and Population]
6	Describe the intervention(s) that will be covered by the COS	INTRODUCTION AND METHODS AND ANALYSIS [Scope – Intervention]
7	Describe the setting(s) that will be covered by the COS	INTRODUCTION AND METHODS AND ANALYSIS
8	Indicate the COS study registration details and registry name. If not yet registered indicate the intended registry	REGISTRATION DETAILS
9	Describe any study oversight committees	STEERING COMMITTEE AND PATIENT AND PUBLIC INVOLVEMENT
10	Describe sources of funding, role of funders	FUNDING STATEMENT
11	Describe any potential conflicts of interest within the study team and how these will be managed	CONFLICTS OF INTEREST
12	Describe the stakeholder groups to be involved in the COS development process and the rationale for their involvement	METHODS –STAKEHOLDERS
13	Describe the eligibility criteria for individuals from each stakeholder group	METHODS –STAKEHOLDERS
14	Describe how individuals of each stakeholder groups will be identified	METHODS –STAKEHOLDERS
15	Describe how individuals of each stakeholder group will be chosen from within the stakeholder group	METHODS –STAKEHOLDERS

For peer review only

16	Describe how many planned individuals within each stakeholder group will be invited to participate in the consensus process	METHODS –STAKEHOLDERS [Participants – Sample Size]
17	Describe how individuals will be invited to take part in the consensus process	METHODS –STAKEHOLDERS
18	Describe the information sources that will be used to identify the list of outcomes. Outline the methods or reference other protocols/papers.	METHODS – SYSTEMATIC REVIEW AND QUALITATIVE INTERVIEWS
19	Describe how outcomes may be dropped/combined, with reasons	METHODS
20	Describe the methods to identify outcome descriptor terms	METHODS
21	Describe the plans for how the consensus process will be undertaken	METHODS
22	Describe what information will be presented to participants at the start of the consensus process	METHODS
23	Describe what each participant will be asked to do at each stage of the consensus process	METHODS
24	Describe how the participants will receive any feedback during the consensus process	METHODS
25	Describe how non-response (or partial response) will be handled during the consensus process	METHODS
26	Describe how the study material will be made patient friendly and understandable (if relevant)	METHODS
27	Describe the consensus definition	METHODS
28	Describe the procedure for determining how outcomes will be added/combined/dropped from consideration during the consensus process	METHODS
29	Describe how outcomes will be scored and summarised	METHODS
30	Describe how the response rate will be maximised	METHODS
31	Describe how attrition bias will be assessed	METHODS
32	Describe any software that will be used during the consensus process and to analyse the results	METHODS
33	Describe any plans for obtaining research ethics committee / institutional review board approval in relation to the consensus process (if relevant)	ETHICS/ DISSEMINATION
34	Describe how informed consent will be obtained (if relevant)	ETHICS/ DISSEMINATION & METHODS
35	Describe any details about how the confidentiality of data collection will be preserved during the consensus process (if relevant)	METHODS

For peer review only

For peer review only

A)The parent experience

I'd like you to tell me your story with as much detail as possible. Then I may have some extra questions if you have not covered them already. We would like to find out your experience and we are particularly interested in how your loss has affected you/and your partner's health.

1) The diagnosis of the stillbirth

- Can you take me back to the beginning of you story, perhaps start from when you first found out you were pregnant?

2) The time between diagnosis and birth (If applicable)

- What happened after you found out you your baby had died e.g. did you go home/stay in hospital/be induced?

3) The birth of your baby

- What was your experience of giving birth to your baby?
- How do you feel about the way you gave birth now?

4) Your stay in hospital

- What happened after your baby was born?

5) Memory Making

- How did you spend time with your baby? If you were unable to spend time with baby, then why not?

Examples include: did you see or hold the baby, did you take photos, foot prints

- How did it make you feel at the time?
- What do you feel about the experience now?
- Which memories are the most meaningful to you now?

6) Post mortem & hospital tests

- What hospital tests did you or your baby have afterwards?
- What information did you learn from the post mortem and/or additional tests?
- How did you feel about your choice at the time & how do you feel about it now? (If did or did not have PM)

7) The review process by the hospital (perinatal mortality review)

- What was your experience of the hospital review process of you and your baby's case?
- Were the involved in the case?
- If parental engagement In review – how did it affect them?

8) The funeral

- What information & support were you given about the baby's funeral?
- If had funeral, can you tell me more about it
- How did that make you feel at the time/how do you feel about it now?

9) When you went home

- Can you tell me about how you made the decision about going home?
- What happened in the days/weeks after you went home?
- How did you feel when you went home?

10) Follow up care by healthcare professionals (e.g hospital consultant, midwife, GP, anyone else?)

For peer review only

- What follow up care did you receive from the hospital e.g. follow up appointment with consultant, bereavement midwife?
- What advice were you given in your follow up appointment about becoming pregnant again?
  - *Were you told how long you should wait before becoming pregnant again (if not discussed)? What will the care be like? What will the birth be like? What were your thoughts about this?*
- What was the contact with the GP/community midwife like?
- What was the impact of the healthcare professionals care on you?

11) Additional care

- Following your experience did you seek any further professional advice or care?
  - *Examples include second opinion, counselling*
- If yes, how did you feel about this care?
- What was the impact of counselling on you? May consider benefits and harms?
- Were there any other ways/methods you dealt with the stillbirth?
  - *Examples could include exercise, mindfulness, charity work, yoga, new hobby, support groups*

12) Support groups

- Did you seek any help from support groups?
- How did that impact you?
- Did you seek any support from online communities?

13) Plans for future pregnancies

- What were your thoughts about becoming pregnant again?

What influenced your decision? Did you seek any alternative advice? What were the sources of your information?

B) Outcomes

1a) Impact on next pregnancy (if applicable)

- Have you had any more pregnancies since? Tell me about your next pregnancy
- How did your previous experience affect your next pregnancy?
- How were you looked after in your next pregnancy?
- Examples: were you treated differently by medical professionals during antenatal care? did you have any extra care, appointments, scans or tests? Was the type of birth different?
- How did becoming pregnant affect you and/or your partner?
- How did being pregnant affect your health? (physical & mental)
- What support (psychological) was offered during pregnancy and after birth?
- Were there any complications in the subsequent pregnancy?
- Did the subsequent pregnancy have an effect on other areas of your life?

1b) If no further pregnancies:

- How did not becoming pregnant again affect you?
- Did you seek any fertility treatment & If yes, could you tell me about it and how did it effect you?

2) Impact of experience on physical & mental health

Opening statement: In this part of interview I will ask about you and your partners health including the impact the stillbirth had on your physical and mental health

- How do you think your experience affected your health in the short term (physical and mental)?
- How do you think your experience affected your health in the long term (physical and mental)?

For peer review only

If does not mention:

Has your experience affected your mental health?
Have you had low mood/anxiety/PTSD?
How has your experience affected your physical health?
Has is it affected your sleep?
Has your experience affected your body image/self-esteem?

- Did you do anything to improve your health after the experience? What were you encouraged to do?
- Could there have been anything done to minimise the impact on you and your health?
- Have any new medical conditions emerged since the stillbirth? Have you had any treatment/therapy?
- What medications have you taken (if any) following the stillbirth?

14) Relationships

- How has your experience affected your relationships?
  - Your partner (weeks, months, years); Your family (weeks, months, years); Your friends (weeks, months, years)
- What was the impact of new babies on you?

15) Relationship with children (if applicable)

- How has your experience affected your relationship with your children?
- Has your experience affected bonding/parenting with your children?
- Have your children needed to have any additional support?

16) Communication

- How did you tell people about your stillbirth?
- How did they respond and how did it affect you?
- What was the impact of social media/media/news stories on you?

17) Employment - going back to work/Finances

- How did you break the news to your colleagues?
- How did your experience affect your job? (if applicable)
- How did the response of your employer affect you (and/or your decision to return to work)?
- How did your experience affect your finances? How did finances affect your decision making?
- If money was no issue, when would have been the right time to return to work in your opinion?

18) Outcomes to develop core outcome set

- If we were going to improve care or research after stillbirth what would be important in your opinion to measure to see if the care worked? Examples include mental health, physical, return to work
- If we were going to improve health after stillbirth what aspect of health would be important to you to improve?
- If unable to answer: How would you improve about the care you received. If that care was improved how would that affected your life and health?

19) Key messages to parents & healthcare professionals

Opening statement: Bearing In mind parents might receive this information whilst in hospital/preparing to go into hospital or newly discharged…

- What would be your advice to parent who might be going through a similar experience?

- What would be your overall key messages to healthcare professionals looking after parents who experience stillbirth?

For peer review only

Supplementary material 3: Think aloud topic guide

Interview Guide

Harmonising outcomes for research and care after stillbirth (The iCHOOSE Study)

Think-aloud interviews for the development of a core outcome set questionnaire

Introduction to study

These interviews aim to find out about how you go about completing a questionnaire to develop a core outcome set. You will be asked to provide a running commentary on how you rate the importance of individual outcomes. We are particularly interested in how you understand the questions, how difficult or easy it is to rate the outcomes, the wording of the outcomes and any changes you would make to the questionnaire. We are also interested in whether there are additional outcomes that should be included in the questionnaire.

If at any time you don't want to continue the interview, you are free to tell me that you wish to stop, and we can either take a break or you can stop completely. It's fine for you to do that.

Interview Instructions

Participants will be read the following instructions, adapted from Green and Gilhooly (1996) and French et al. (2007):

We are interested in how people complete the following questionnaire. This questionnaire will ask you to rank outcomes on how important they are to you to measure in research and evaluating care after stillbirth. We want to check that people understand the questions in the way that we meant them. To do this, I am going to ask you to 'think aloud' as you complete the questionnaire. What I mean by 'think aloud' is that I want you to tell me everything you are thinking as you read each question and decide how to answer it. I would like you to talk aloud constantly. I don't want you to plan out what you say or try to explain to me what you are saying. Just act as if you are alone in the room speaking to yourself. If you are silent for any long period of time, I will ask you to talk or ask you question to help you. Please try to speak as clearly as possible, as I shall be recording you as you speak. Do you understand what I want you to do?

Interview question probes (Adapted from Collins (2003), French et al (2007) and McCorry (2013))

General

- Tell me what are you thinking?
- How did you go about rating that outcome?
- How easy or difficult did you find this outcome to rate?
- Would you like to make any changes to this question/outcome?

For peer review only

Comprehension

- What does that outcome mean to you?
- What did you understand by this outcome?
- Are there any problems with the wording of this outcome?

Retrieval

- How did you calculate your answer?
- Is this outcome relevant to a particular time period that you can relate to?
- How applicable is this outcome to your individual circumstances?

Confidence judgement

- How sure of your answer are you?

Response

- How did you feel about answering this question?

Additional outcomes

- Would you like to include any other additional outcomes?

For peer review only

Supplementary material 4: Table of changes for think aloud interviews and questionnaire development

Delphi Questionnaire for core outcome set for research after stillbirth					
Negative Comments	***Positive Comments***	***Possible Change***	***Reason for change***	***Agreed change***	***MoScoW***

For peer review only

For peer review only

BMJ Open

Protocol for the development of a core outcome set for stillbirth care research (iCHOOSE Study)

Journal:	*BMJ Open*
Manuscript ID	bmjopen-2021-056629.R1
Article Type:	Protocol
Date Submitted by the Author:	08-Nov-2021
Complete List of Authors:	Bakhbakhi, Danya; University of Bristol Medical School, Translational Health Sciences<br>Fraser, Abigail; University of Bristol Medical School, Population Health Sciences<br>Siasakos, Dimitris; UCL, UCL EGA Institute for Women's Health<br>Hinton, Lisa; THIS Institute<br>Davies, Anna; University of Bristol Medical School, Translational Health Sciences<br>Merriel, Abi; University of Bristol Medical School, Population Health Sciences<br>Duffy, James; King's Fertility, The Fetal Medicine Research Institute<br>Redshaw, Maggie; NPEU, Department of Population Health<br>Lynch, Mary ; University of Bristol Medical School<br>Timlin, Laura; North Bristol NHS Trust, Women & Children's Health<br>Flenady, Vicki; Mater Research Institute-University of Queensland,<br>Heazell, Alexander; University of Manchester, Maternal and Fetal Health Research Centre<br>Downe, Soo; University of Central Lancashire, research in childbirth and health<br>Slade, Pauline; University of Liverpool, Psychological Sciences<br>Brookes, Sara; University of Birmingham<br>Wojcieszek, Aleena; Mater Research Institute - The University of Queensland, Centre of Research Excellence in Stillbirth<br>Murphy, Margaret; University College Cork National University of Ireland, Nursing and Midwifery<br>de Oliveira Salgado, Heloisa; University of São Paulo<br>Pollock, Danielle; The University of Adelaide, Public Health<br>Aggarwal, Neelam ; Post Graduate Institute of Medical Education and Research<br>Attachie, Irene; University of Health and Allied Sciences School of Public Health<br>Leisher, Susannah; International Stillbirth Alliance, ;<br>Kihusa, Wanijiru; Still a mum<br>Mulley , Kate ; Sands<br>Wimmer, Lindsey ; Star Legacy Foundation<br>., UK iCHOOSE parent involvement group; University of Bristol Faculty of Health Sciences<br>Burden, Christy; University of Bristol Medical School, Translational Health Sciences
Primary Subject	Obstetrics and gynaecology

Heading:	
Secondary Subject Heading:	Research methods
Keywords:	OBSTETRICS, QUALITATIVE RESEARCH, Quality in health care < HEALTH SERVICES ADMINISTRATION & MANAGEMENT, Protocols & guidelines < HEALTH SERVICES ADMINISTRATION & MANAGEMENT, Fetal medicine < OBSTETRICS, Maternal medicine < OBSTETRICS

SCHOLARONE™
Manuscripts

Title

Protocol for the development of a core outcome set for stillbirth care research (iCHOOSE Study)

Danya Bakhbakhi, University of Bristol, danya.bakhbakhi@bristol.ac.uk
Abigail Fraser, University of Bristol, abigail.fraser@bristol.ac.uk
Dimitris Siasakos, University College London, d.siasakos@ucl.ac.uk
Lisa Hinton, University of Cambridge, lisa.hinton@thisinstitute.cam.ac.uk
Anna Davies, University of Bristol, anna.davies@bristol.ac.uk
Abi Merriel, University of Bristol, abi.merriel@bristol.ac.uk
James M N Duffy, King's Fertility, james.duffy3@nhs.net
Maggie Redshaw, University of Oxford, maggie.redshaw@npeu.ox.ac.uk
Mary Lynch, University of Bristol, mary.lynch@nbt.nhs.uk
Laura Timlin, North Bristol NHS Trust, laura.timlin@nbt.nhs.uk
Vicki Flenady, The University of Queensland, vicki.flenady@mater.uq.edu.au
Alexander Heazell, University of Manchester, alexander.heazell@manchester.ac.uk
Soo Downe, University of Central Lancashire, sdowne@uclan.ac.uk
Pauline Slade, University of Liverpool, pauline.slade@liverpool.ac.uk
Sara Brookes, University of Birmingham, s.t.brookes@bham.ac.uk
Aleena Wojcieszek, The University of Queensland, aleena.wojcieszek@uqconnect.edu.au
Margaret Murphy, University College Cork, mgt.murphy@ucc.ie
Heloisa de Oliveira Salgado, University of Sao Paulo, hellosalgado@gmail.com
Danielle Pollock, JBI, University of Adelaide, danielle.pollock@adelaide.edu.au
Neelam Aggarwal, Post Graduate Institute of Medical Education and Research, Chandigarh, India, drneelamaggarwal@gmail.com
Irene Attachie, University of Health and Allied Sciences, Ghana, iattachie@uhas.edu.gh
Susannah Hopkins Leisher, International Stillbirth Alliance, shleisher@aol.com
Wanjiru Kihusa, Still a Mum, Kenya, wanjiru@stillamum.com
Kate Mulley, Sands Charity, United Kingdom, kate.mulley@sands.org.uk
Lindsey Wimmer, Star Legacy Foundation, lindsey@starlegacyfoundation.org
UK iCHOOSE parent involvement group

Christy Burden, University of Bristol, christy.burden@bristol.ac.uk

Corresponding Author: Dr Danya Bakhbakhi, danya.bakhbakhi@bristol.ac.uk, Centre for Academic Women's Health, University of Bristol, Chilterns, Women's Health, Southmead Hospital, Westbury on Trym, Bristol BS10 5NB, United Kingdom, 0117 950 5050

Keywords

Stillbirth, Consensus, Pregnancy, Research design, Core outcome set

Word count

4929

Abstract

For peer review only - http://bmjopen.bmj.com/site/about/guidelines.xhtml

Introduction

Stillbirth is associated with significant physical, psychosocial and economic consequences for parents, families, wider society and the healthcare system. There is emerging momentum to design and evaluate interventions for care after stillbirth and in subsequent pregnancies. However, there is insufficient evidence to inform clinical practice compounded by inconsistent outcome reporting in research studies. To address this paucity of evidence, we plan to develop a core outcome set for stillbirth care research, through an international consensus process with key stakeholders including parents, healthcare professionals and researchers.

Methods and analysis

The development of this core outcome set will be divided into five distinct phases: 1) Identifying potential outcomes from a mixed-methods systematic review and analysis of interviews with parents who have experienced stillbirth; 2) Creating a comprehensive outcome long-list and piloting of a Delphi questionnaire using think-aloud interviews; 3) Choosing the most important outcomes by conducting an international two-round Delphi survey including high-, middle- and low- income countries; 4) Deciding the core outcome set by consensus meetings with key stakeholders; and 5) Dissemination and promotion of the core outcome set. A parent and public involvement panel and international steering committee has been convened to co-produce every stage of the development of this core outcome set.

Ethics and dissemination

Ethical approval for the qualitative interviews has been approved by Berkshire Ethics Committee REC Reference 12/SC/0495. Ethical approval for the think-aloud interviews, Delphi survey and consensus meetings has been awarded from the University of Bristol Faculty of Health Sciences Research Ethics Committee (Reference number:116535). The dissemination strategy is being developed with the parent and public involvement panel and steering committee. Results will be published in peer-reviewed speciality journals, shared at national and international conferences and promoted through parent organisations and charities.

Registration details

COMET Initiative registration number 775
PROSPERO registration number CRD42018087748

Article summary

STRENGTHS AND LIMITATIONS OF THIS STUDY

- Using robust and transparent methodology, this will be the first core outcome set developed for use in stillbirth care research, which will ultimately improve evidence synthesis in this field and could reduce research wastage.

For peer review only

For peer review only - http://bmjopen.bmj.com/site/about/guidelines.xhtml

- In-depth qualitative interviews with parents will enable the identification of novel and parent-important outcomes not identified from the systematic review.
- Parent representation is a strength of this study; we are including bereaved parent stakeholders at every stage of the development, co-producing the research with a parent involvement panel, and have international parent representation within the project steering committee.
- Qualitative interviews (in Stage 1) include UK parents only, however, to help mitigate this limitation and increase the generalisability of the results, we are triangulating our findings with outcomes identified in the systematic review of global literature along with recuiting international stakeholders for the think aloud interviews, Delphi survey and consensus meetings.
- Due to funding limitations and translation costs, the Delphi survey and consensus meetings will be conducted in the English language only, however, future research will endeavour to validate the core outcome set in languages other than English.

Introduction

Worldwide it is estimated that there are 2 million stillbirths every year[1]. Stillbirth is associated with significant physical, psychosocial, health and economic costs for parents, their families, wider society and the healthcare system[2,3,4]. In a subsequent pregnancy, a history of stillbirth has been shown to be associated with higher frequencies of adverse clinical outcomes, including increased risk of stillbirth recurrence, antenatal complications, mental health concerns and impact on subsequent children[5,6,7,8.] The negative consequences of stillbirth are widespread and long-lasting; therefore, it is important to invest in high-quality research to enable healthcare professionals and researchers to deliver the best care for affected families.

Several care-related interventions are available to minimise the negative impact of stillbirth. These interventions can be implemented from the immediate identification of a stillbirth to when parents are discharge from hospital to the community or in a subsequent pregnancy. Examples include, supporting parents' choices around birth and afterwards, offering opportunities for parents to make memories with their baby, support with post-mortem investigation decision making, engagement of the parents in the perinatal mortality review process[9–12], bereavement care from healthcare professionals[13], counselling and specialist care in subsequent pregnancies[14]. Yet very little is known about the effectiveness of these interventions [15].

There is momentum to research, design and evaluate interventions to improve care for parents following stillbirth and in any subsequent pregnancies[15,16,17.] However, systematic reviews suggest few methodologically rigorous studies exist to inform clinical practice and their results cannot be synthesised quantitatively due to a high degree of heterogeneity of outcome reporting[15,17,18]. In 2018, a Cochrane review on care prior to and during subsequent pregnancies following stillbirth for improving outcomes, found insufficient and inconsistent evidence to inform clinical practice[17]. The authors of this review concluded that it is important to have consistency in data collection across all future trials and this may be facilitated by a core outcome set for stillbirth care research[17].

For peer review only - http://bmjopen.bmj.com/site/about/guidelines.xhtml

A core outcome set is a consensus-derived minimum set of outcomes that should be measured and reported in all research studies of a specific disease or trial population[19]. It does not preclude the measurement of additional specific outcomes; however, a minimum set of outcomes will allow higher quality of evidence to identify the most effective interventions and care packages offered. A recent web-based survey of healthcare professionals, researchers and advocates identified the development of a core outcomes set for stillbirth (and recurrent stillbirth) research as one of the top five priority research topics to inform clinical practice for the care of families following stillbirth[20]. Currently there are no available core outcome sets published for stillbirth care research (i.e. research focusing on care after a stillbirth is identified) https://www.comet-initiative.org/Studies.

The inclusion of patients in the development of a core outcome set is paramount as they are the key stakeholders in the research outcomes. Inclusion of parents can lead to a widening of the research agenda, identifying important patient reported outcomes and recognising previously neglected patient outcomes that matter to those who experience stillbirth[21]. There is a need to develop and evaluate evidence-based interventions using outcomes that directly relate to bereaved parents' experiences. To enable this, it is essential to establish a minimum set of outcomes that includes parents and relevant stakeholders in the development process. If applied in clinical trials, a core outcome set for stillbirth care research developed with stakeholder input, will provide a tool to give consistency in outcome measurement, minimise reporting bias, and allow for direct comparison of interventions and care across research studies. This could lead to better evidence being produced to improve clinical decision making in the future.

Aim and objectives

AIM

The iCHOOSE study aims to develop a minimum set of outcomes that should be evaluated and reported in all future stillbirth care research in high-, middle- and low- income country settings, through an international consensus process of key stakeholders including parents, healthcare professionals, researchers and charity representatives.

OBJECTIVES

1. To investigate what outcomes are reported in existing studies assessing the impact of stillbirth on parents.
2. To investigate parental experiences following stillbirth and identify important outcomes for bereaved parents not reported in the scientific literature.
3. To pilot and develop a Delphi questionnaire, using think-aloud interviews.
4. To achieve international consensus on a core outcome set for stillbirth care research using a Delphi survey technique and stakeholder consensus meetings.
5. To disseminate and promote the core outcome set for stillbirth care research.

Methods and analysis

For peer review only - http://bmjopen.bmj.com/site/about/guidelines.xhtml

There is no standardised way to develop a core outcome set[21]. The COMET initiative has collated methodological resources to assist with the development of the core outcome set including a systematic review outlining the issues to consider[21–24]. COMET resources, including the COMET Handbook: version 1.0 and reviewed published core outcome sets have been used to inform the study design[23–30]. This study is prospectively registered on the COMET website https://www.comet-initiative.org/studies/details/775. The Core Outcome Set-STAndards for Development (COS-STAD) and the Core Outcome Set-STAndardised Protocol Items (COS-STAP) have been followed in the planning of the methods of this core outcome set project[31,32]. **See Supplementary material 1: Core Outcome Set-STAndardised Protocol Items (COS-STAP) Checklist for the iCHOOSE Study.** The final core outcome set will be reported in accordance with the Core Outcome Set-STAndards for Reporting statement (COS-STAR)[33].

SCOPE OF THIS CORE OUTCOME SET

Health condition and population

The core outcome set will be applicable to families who have experienced a stillbirth in a singleton or multiple pregnancy. We will aim for this core outcome set to be applicable to all countries internationally including high-, middle- and low- income countries. The definition of stillbirth varies internationally and therefore the gestation will be dependent on the study setting. It is our intention that this core outcome set could be applied to stillbirths from at least 20 weeks' gestation, including antepartum and intrapartum stillbirths from any cause including due to a congenital abnormality. We will set exclude outcomes related to the termination of pregnancy and neonatal death population.

Interventions

The core outcome set will be relevant to all stillbirth care research. Stillbirth care research includes the care that parents (and families) receive after a stillbirth has been identified. The core outcome set will not be limited by the type of intervention or the setting in which it is delivered. It will cover all medical and psychosocial interventions and care parents are offered following a stillbirth and in a subsequent pregnancy[15,17]. **See Figure 1: Types of interventions after stillbirth that should be evaluated using outcomes identified in the core outcome set.**

Context

The core outcome set will be developed for use in all stillbirth care research (e.g.randomised controlled trials, observational studies and systematic reviews). It is also anticipated that it could be utilised in the evaluation of clinical practice guidelines, care pathways for bereaved parents and training for healthcare professional[34].

PATIENT AND PUBLIC INVOLVEMENT

Parent perspectives are integral to every stage of the development, including the input into this protocol, the systematic review, qualitative interviews, Delphi survey, consensus meeting and dissemination of results. A parent involvement panel has been established and training is being provided using methods exemplified by

For peer review only - http://bmjopen.bmj.com/site/about/guidelines.xhtml

NIHR INVOLVE. The parent involvement panel have also co-designed the parent animation video to aid recruitment https://vimeo.com/292143259/f2edb109dd.

STEERING COMMITTEE

An international expert steering committee including healthcare professionals, parents with a lived experience of stillbirth, charity representatives and researchers with diverse expertise has been convened to guide the research design, recruitment and development of the core outcome set. This group has stakeholder representation from Europe, Australia, North America, South America, Africa and Asia.

COLLABORATIONS

We have established the International Collaboration for Harmonising Outcomes fOr Stillbirth research and carE (iCHOOSE) initiative. The iCHOOSE collaboration aims to develop a core outcome set for stillbirth care research with the overall aim of improving outcomes for parents and the wider family. This collaboration is endorsed by the Core Outcomes in Women's Health (CROWN) initiative; the Medical Sociology and Health Experiences Research Group, University of Oxford; the National Stillbirth Centre for Research Excellence, Australia, The Stillbirth and Neonatal Death Charity (Sands); Tommy's National Centre for Maternity Improvement, Twins Trust, Star Legacy Foundation and International Stillbirth Alliance.

STUDY OVERVIEW

The study will be divided into five distinct stages. **See Figure 2: iCHOOSE Study overview**

STAGE 1: IDENTIFYING POTENTIAL OUTCOMES

Systematic review: What outcomes have been reported?

Previously reported outcomes and associated outcome measurement tools relevant to stillbirth care research are being identified through a systematic review of the literature. Our systematic review process has been prospectively registered on PROSPERO International prospective register of systematic reviews (CRD42018087748). The electronic databases MEDLINE, PubMed, Embase, Scopus, Amed, BNI, CINAHL, PsycINFO Cochrane Register of Controlled Trials will be searched from 1998 to present**.** Reference lists of extracted articles will also be searched. We will include all randomised trials, observational and qualitative studies that report an outcome following stillbirth. Case reports, editorials, review articles, abstracts and grey literature will be excluded. Studies including mothers, fathers, children, siblings, and grandparents experiencing a stillbirth in a singleton or multiple pregnancy will be included. Studies will not be excluded based on the gestational definition of stillbirth, as the definition varies between jurisdictions. Titles, abstracts and full texts of studies will be screened independently by two review authors using Covidence systematic review software[35]. Disagreements will be resolved through a third reviewer.

For peer review only - http://bmjopen.bmj.com/site/about/guidelines.xhtml

A standardised, pre-piloted electronic data extraction form has been developed to extract data. Data will be extracted in duplicate and includes basic publication details (including author and date of publication); study setting; study population; details of intervention (if applicable); study methodology; outcomes measured verbatim, their definition (if stated), their relevant outcome measurement tool (if applicable) and whether the tool is validated for that cultural context and if parents and members of the public were involved in the outcome selection. A sequential explanatory approach will be undertaken i.e., outcomes from quantitative studies will be extracted initially followed by outcomes reported in the qualitative literature. This will be done to compare, and contrast outcomes reported in the qualitative literature. A comprehensive inventory of outcomes reported will be developed from the data extraction The systematic review will be reported using the Preferred Reporting Items for Systematic Reviews and Meta-Analyses (PRISMA) guidelines[36].

Qualitative interviews: What outcomes are important to parents?

Capturing patient perspectives is crucial in the development of a core outcome set as they often identify outcomes not considered by other stakeholders or within the literature [37]. Parents with a lived experience of stillbirth in the United Kingdom (UK) will be recruited to participate in qualitative interviews through Sands, National Health Service Hospital Trusts, the Twins Trust, bereavement support groups, the parent involvement panel and snowballing through personal contacts of the research team and the parent involvement panel. To ensure diverse opinions participants will be purposively sampled for maximum variation. Participants will include mothers and fathers/partners from a wide range of social, ethnic, and cultural backgrounds who have experienced a stillbirth at a range of gestations and time periods since the stillbirth occurred. Parents who have a personal history of a stillbirth at more than 24 weeks' gestation (UK definition), at least six months prior to the study would be eligible to participate. This definition was chosen as recent research has focused on parents' experiences of care following the death of a baby in pregnancy between 20 and 24 weeks in the UK[38]. The findings of this research will be incorporated into the systematic review findings. Furthermore, as we are only recruiting UK parents, we plan to triangulate the data with outcomes extracted from the qualitative data from the systematic review. Parents will be interviewed individually or jointly, according to preference. The number of parents recruited will depend on when theoretical saturation is reached (i.e. when no new themes emerge) [39].

With informed consent, semi-structured interviews with parents will be conducted in either parents' homes, a suitable private location of their choice or via Zoom teleconference software. A researcher with training in qualitative interview methods will conduct the interviews (DB) supported by an experienced qualitative researcher (LH). The interviews will invite parents to narrate their lived experienced of stillbirth. However, an interview topic guide has also been developed in consultation with the parent involvement panel and guided by the literature review **(See Supplementary material 2: Interview topic guide).** The interviews will aim to answer the following questions: 1) What are parents' experiences following stillbirth? 2) What issues (outcomes) are important to parents after they have experienced a stillbirth? 3) What outcomes do parents think are important to measure so stillbirth care can be improved through research? Interviews will be audio and/or video recorded and transcribed verbatim. Stillbirth is a sensitive topic, and it is possible parents may experience distress during the interviews; should this happen, they will be offered the opportunity to pause the interview and, if they

For peer review only - http://bmjopen.bmj.com/site/about/guidelines.xhtml

choose, to stop it completely. They will be signposted to support from their own healthcare provider or community support services.

Data collection and analysis will be guided by an iterative approach, allowing data analysis of early interviews to enrich data collection of later interviews. Following a familiarisation process, data will be coded blinded and in duplicate. Each line of the transcript will be coded systematically, identifying outcomes anchored in the words of the participant. Using an inductive approach, a codebook will be generated, and the data will be managed using NVivo software which will help to organise emergent themes. A constant comparative method will be adopted, whereby transcripts will be re-read, and codes compared with every other occurrence in the interviews. Data will be analysed and conceptualised into broader categories using the 'One sheet of paper' technique[40] and the DIPEx (personal experiences of health experiences and illness) techniques for coding[40]. This approach has been taken to generate a deeper understanding and meaning of the outcomes, in the context of the lived experience of stillbirth, using the detail-rich interview transcripts. A collaborative approach will be taken with the analysis whereby emergent themes and codes will be developed iteratively with input from members of the project steering committee. The Consolidated criteria for reporting qualitative research (COREQ) checklist will be used to report the findings of the qualitative interviews[41].

STAGE 2: CREATION OF OUTCOME LONG-LIST AND PILOT WITH THINK ALOUD INTERVIEWS

Creation of outcome long-list

A comprehensive outcome inventory will be developed from all the outcomes identified in the data extraction of the systematic review and analysis of the qualitative interviews. As an initial step, we will group similar definitions (extracting the wording description verbatim) under the same outcome name[23]. Outcomes will then be grouped into outcome domains or categories to classify the broad aspects of the effects of interventions or care[23]. The outcomes will be organised into outcome categories using an adapted taxonomy that has been developed for outcomes in medical research to help improve knowledge discovery[42]. Each verbatim outcome definition will be categorised to an outcome name and mapped to a domain independently by two researchers from multi-professional backgrounds (a health care professional and a health service research methodologist) to provide transparency. Any differences will be resolved by consulting a senior member of the research team.

Consideration will be given to the order of questions and the number of items as previous research has demonstrated that question order could affect response rates and actual responses to question items[43]. The final outcome long-list will be reviewed by the steering committee and parent involvement panel. Furthermore, with input from the parent involvement panel plain language definitions will be developed for each outcome item.

Pilot and think-aloud interviews

The questionnaire items and response scale format will be piloted using the think-aloud approach to ensure the ease of completion, readability, understandability and acceptability by stakeholders prior to recruitment[23,44,45].

For peer review only - http://bmjopen.bmj.com/site/about/guidelines.xhtml

It will also be used to refine the long-list of outcomes. The think-aloud method has been used by other core outcome set developers to improve their questionnaire design[45–48,49]. We will examine how parents and other stakeholders interpret the outcome labels and definitions, check they understand how to complete the nine-point Likert rating scale and identify problems[23]. Participants will think aloud as they work through the draft Delphi and provide a running commentary on their thoughts on rating of outcomes[23]. The interviewer will use open-ended cognitive probes as described in the interview guide **(See Supplementary material 3: Think aloud topic guide).** The probes will ascertain comprehension, retrieval, confidence judgement and responses to questions[45]. We will also determine the length of time it takes to complete the survey to ensure response fatigue is minimal.

Interviews will be face-to-face or via Zoom teleconferencing and will be audio recorded once informed consent has been obtained. Transcribed interviews will be coded, by two independent researchers according to a framework of think-aloud categories[50]. The coded comments will be subsequently tabulated in a 'table of changes' and for each outcome to provide a transparent method of recording suggestions **(See Supplementary material 4: Table of changes for think aloud interviews and questionnaire development).** Suggested changes in wording, reasons for change and agreed changes will be documented providing transparency in the questionnaire development. This approach has been used in think-aloud interviews within the Person-Based Approach to intervention development[51,52]. An iterative approach will be adopted; we will revise the questionnaire following analysis of an initial sample of think aloud interviews, conduct further interviews, and revise the questionnaire until data saturation and no further changes are indicated. We estimate that we will interview approximately 12 to 15 stakeholders. Following these interviews, the final Delphi questionnaire will be produced.

STAGE 3: INTERNATIONAL DELPHI SURVEY

The core outcome set will be determined using a modified Delphi method. The Delphi methodology has been used to allow stakeholders with expert knowledge on a particular subject to achieve convergence of opinion on the importance of different outcomes using sequential questionnaires or face-to-face meetings[23]. Responses for each outcome will be summarised and fed back anonymously in the following questionnaire round. Participants will be able to consider the responses of others and their previous response before re-scoring each item; this has the benefit of allowing participants to review previous round results independently, with the overall aim to achieve consensus.

Selection and recruitment of stakeholders

Representatives from all stakeholder groups will be invited to participate in the think-aloud interviews, the Delphi survey and consensus meetings. Stakeholders will include two main groups: parents with a lived experience of stillbirth and professionals. The professional stakeholder group will include healthcare professionals caring for parents who have experienced stillbirth (e.g. obstetricians, midwives, general practitioners, sonographers, psychiatrists, psychologists and doulas), researchers, bereavement charity representatives and stillbirth advocates. Due to translation costs and financial limitations of the study, non-

For peer review only - http://bmjopen.bmj.com/site/about/guidelines.xhtml

English speakers will be excluded. A stakeholder recruitment sampling frame will be created to ensure there is maximum variation in the sample.

As stillbirth occurs globally, participants will be sought through an international network of parent support groups, organisations, professional associations and charities, including from high, low- and middle- income countries. We will aim to achieve representation from most continents including Europe, Africa, Asia, Australia, North America and South America. We will aim to recruit a diverse range of mothers and fathers/partners who have experienced a stillbirth at a range of gestations and time periods since the stillbirth occurred. Family members of parents who experience stillbirth, for example grandparents, siblings or other immediate family member will also be eligible to participate. Parents will be identified via charity support groups, social media, and the International Stillbirth Alliance. We will work with international collaborators in participating countries to use websites and social media that are most relevant to parents that we wish to approach. Healthcare professionals will be identified via email distribution lists using links with the Royal College of Obstetricians and Gynaecologists, the Royal College of Midwives, the International Stillbirth Alliance, the British Psychological Society (counselling, health psychology and clinical psychology divisions), Royal College of General Practitioners and British Association for Counselling and Psychotherapy. Researchers will be identified through authors of papers in the systematic review, and research networks.

Sample size

There are no generally accepted guidelines for the optimal size to achieve a consensus in Delphi Studies. Decisions about on how many individuals to include in a Delphi process is pragmatic, and not based on statistical power[23,53]. Careful consideration will be made to sample stakeholders with a breadth of experience. For the Delphi survey, a minimum of 100 participants per stakeholder group (100 parents and 100 professionals) will be recruited to account for a 20% drop-out rate[54,55]. This estimate is based on the typical response rate found from a review of published and ongoing studies that included Delphi to develop a core outcome set[54] We will use evidence-based methods for maximizing recruiting and retaining participants between rounds, for example, direct personalised email invitations, promotional animation and demonstration videos for each round of the Delphi and adopting a minimum waiting time between rounds one and two[54–56].

Delphi Survey

Respondents will be invited to complete two sequential rounds of the Delphi survey via email. Study data will be collected and managed using REDCap (Research Electronic Data Capture) tools hosted at the University of Bristol[57]. REDCap is a secure, web-based application designed to support data capture for research studies, providing: 1) an intuitive interface for validated data entry; 2) audit trails for tracking data manipulation and export procedures; 3) automated export procedures for seamless data downloads to common statistical packages; and 4) procedures for importing data from external sources[57]. Informed consent will be obtained via REDCap from all participants who agree to take part. The data will be analysed using STATA[57].

Participants will be asked to indicate the importance of each outcome using a nine point Likert scale devised by the Grading of Recommendations Assessment Development and Evaluations (GRADE) working group[58]. They will also be given the opportunity to add additional outcomes to be incorporated into round two of the survey. After round one, data will be analysed using descriptive statistics to produce a summary of the results, including

For peer review only - http://bmjopen.bmj.com/site/about/guidelines.xhtml

the presentation of the results in histograms. An anonymous summary of the responses will be fed back to participants according to each stakeholder group in round two of the survey and each participant will receive their own previous scores for round one. Participants will be asked to reflect on the stakeholder group scores and their own score before rescoring each outcome and new outcomes identified by participants from round one. Any outcomes not deemed important by the pre-specified criteria (see below) will be excluded. If a participant does not complete round two of the Delphi survey, their scores from round one will be counted as valid and retained in the study. The rate of missing responses will be reported with the results of the Delphi survey. The round two results will be reviewed by the steering committee to consider the need for a third Delphi survey round.

Consensus definition

A standardised consensus definition will be applied to enable core outcomes to be identified: [1] 'Consensus in' (classify as a core outcome): Over 70% of participants in at least one stakeholder group score outcome 'critical' (score seven to nine) and less than 15% of participants in at least one stakeholder group score outcome 'limited importance' (score one to three). [2] 'Consensus out' (do not classify as a core outcome): Over 70% of participants in at least one stakeholder group score outcome domain 'limited importance' (score one to three) and less than 15% of participants in at least one stakeholder group score outcome domain 'critical' (score seven to nine); or [3] 'No Consensus' (do not classify as a core outcome): Anything else[23,26]. **See figure 3: Consensus definition.**

The rationale for this definition is that for an outcome to be included in the core outcome set, it requires agreement by the majority that it is of critical importance and only a small minority consider it to have little importance. This definition will be reviewed by the steering Committee after Round 1 of the Delphi if a large proportion of outcomes are classified as 'Consensus in'. Possible strategies that could be adopted to be more stringent in the definition could include, having a higher percentage cut-off of stakeholders who need to score an outcome seven to nine to be 'Consensus in' (80% of participants in at least one stakeholder group) or deciding an outcome to be 'critical' only if scored eight to nine. Particular caution will be applied in the review of this definition to ensure that variation in parents' views is not lost between rounds.

STAGE 4: CONSENSUS MEETINGS TO DECIDE THE CORE OUTCOME SET

At least two consensus meetings will take place to discuss the results of the survey and agree the final core outcome set. Stakeholders will be asked if they are willing to participate in the consensus meetings at the end of the Delphi questionnaire and will invited once the analysis of round two has been completed. If a large number of stakeholders are interested in attending the meetings, we will aim to have minimum representation from each continent and each stakeholder group. It is anticipated that these meetings will be either face-to-face or virtually via Zoom teleconferencing software and informed consent will be taken prior to commencement of each meeting. The meetings will be run sensitively by researchers and a bereavement care midwife who are experienced in running research meetings with bereaved parents[9,59]. A representative from the Sands Charity and International Stillbirth Alliance will also be present for the meeting to support parents if required. The initial meeting will take place only with parents. This pre-meeting will allow parents to have the

For peer review only - http://bmjopen.bmj.com/site/about/guidelines.xhtml

equal opportunity to voice their opinions without intimidation or influence from the other stakeholder groups. A sub-set of parent representatives will be invited to the second consensus meeting (and potentially third consensus meeting) with all stakeholder groups.

A modified Nominal Group Technique will be used to further prioritise consensus outcomes[60]. This technique ensures that all participants have the opportunity to provide their perspectives and to hear the views of others. The modified Nominal Group Technique does not rely on statistical power. It is anticipated that eight to ten participants from each stakeholder group will participate in the consensus meetings, as this number has yielded sufficient results in the development of previous core outcome sets[61],[62].

Prior to the meeting attendees will be sent a reminder of their own personal Delphi score. A facilitator will present the results from the earlier rounds according to each stakeholder group. All potential core outcomes reaching the standardised definition for 'Consensus in' will be discussed. Participants in the meeting will be either asked to work individually or split into small groups or pairs to consider the outcomes, including any outcomes that they feel are missing. All the participants are then brought together to discuss each outcome in turn. Each participant will be asked to contribute their opinions on outcomes considered for inclusion in the final core outcome set. With consent of the participants the consensus meetings will be audio and video recorded and minuted.

A further round of voting and discussion will take place with the aim of achieving consensus and ratifying the final core outcome set. Items will be categorised as 'Consensus in – outcome included in the final core outcome set', 'Consensus out – outcome not included in the final core outcome set' or 'No consensus - outcomes for which opinions on inclusion are divided'. This will be facilitated by online, smartphone or electronic keypad technology, allowing for all present to vote anonymously and simultaneously. Outcomes will be rejected where there is again 'No consensus' reached at this stage. The transcribed meeting will be uploaded onto NVivo and analysed using a content analysis to contextualise the decision making around the development of the core outcome set[63].

Identifying outcome measurement tools using the literature

Once the core outcome set is agreed it is important to determine how outcomes should be measured so that the core outcome set can be fully utilised[21,23]. Currently there are no guidelines available to support outcome measurement instrument selection for core outcome sets. Future research will include identifying potential outcome measurement tools for each outcome in the core outcome set from the systematic review. If no outcome measurement tools are identified for a core outcome using this method, this will be acknowledged, and identification and/or development, quality assessment and selection of suitable outcome measurement tools will form part of future research work.

Ethics and dissemination

For peer review only - http://bmjopen.bmj.com/site/about/guidelines.xhtml

STAGE 5: SHARE AND PROMOTE - *Dissemination*

We are aiming for this core outcome to be used in all future stillbirth care research. The dissemination strategy will be developed with the steering committee, the parent involvement panel, and the University of Bristol's Public Engagement Office. A range of methods will be used to raise awareness of the core outcome set and promote its adoption. The results of the systematic review, qualitative interviews, think-aloud interviews, the Delphi process and consensus meetings will be published in peer-reviewed speciality journals. An overview of the core outcome set will be disseminated to the Core Outcomes in Women's Health (CROWN) and Core Outcome Measures in Effectiveness Trials (COMET) initiatives. The results will be presented at national and international scientific conferences of the International Stillbirth Alliance (ISA), Royal College of Obstetricians and Gynaecologists, Royal College of Midwives, International Federation of Gynecology and Obstetrics, British Maternal & Fetal Society, the and the COMET conferences. Furthermore, we will promote a high-level awareness of the study and the core outcome set through social media via parent organisations and charities. Results will also be directly shared with professional associations, relevant university research departments and clinical guideline developers to maximise uptake of the final core outcome set.

Ethics

Ethical approval for the qualitative interviews has been approved by Berkshire Ethics Committee REC Referene 12/SC/0495.

Ethical approval for the think-aloud interviews, Delphi survey and consensus meetings has been awarded from the University of Bristol Faculty of Health Sciences Research Ethics Committee (Reference number:116535).

AUTHOR CONTRIBUTIONS

Study concept: DB, CB, AF, AD, DS, LH, JMND, SB: Study Design: DB, CB, AF, AD, DS, LH, JMND, SB MR, AH, VF, ML, AM, LT, SD, PS, AW, MM, HOS, DP, NA, SL, IA, WK, KM, LW, UKPIG. Drafting of the article: DB. Critical revision of the article for important intellectual content and approval of final manuscript: DB, CB, AF, AD, DS, LH, JMND, SB MR, AH, VF, ML, AM, LT SD, PS, AW, MM, HOS, DP, NA, SL, IA, WK, KM, LW, UKPIG. Study supervision: CB, AF, DS, LH, AD

ACKNOWLEDGEMENTS

The authors thank the UK iCHOOSE parent involvement panel for their involvement in the co-production of this research project: Lisa Thorne, Will Hatton, Carla Mereu Keating, Heather Jane Coombs, Dave Coombs, Michelle Fischer, Ali Fischer, Fraser Morton, Naomi Hepworth.

FUNDING STATEMENT

This report is independent research arising from a doctoral fellowship (DRF) supported by the National Institute for Health Research (NIHR DRF-2017-10-130). The views expressed in this publication are those of the authors

For peer review only - http://bmjopen.bmj.com/site/about/guidelines.xhtml

and not necessarily those of the National Health Service, the National Institute for Health Research, or the Department of Health.

DATA STATEMENT

Not available as protocol only.

COMPETING INTERESTS STATEMENT

Dr Aleena Wojcieszek has received consulting fees from the Sillbirth CRE. There are no other competing interests to declare.

FIGURE LEGENDS

Figure 1: **Types of interventions after stillbirth that should be evaluated using outcomes identified in the core outcome set**
Figure 2: iCHOOSE Study overview
Figure 3: Consensus definition

References

1. Unicef. Unicef Stillbirth data. 2020.
2. Heazell AEP, Siassakos D, Blencowe H, Burden C, Bhutta ZA, Cacciatore J. Stillbirths: economic and psychosocial consequences. Lancet [internet]. Elsevier. 2017;387.
3. Burden C, Bradley S, Storey C, Ellis A, Heazell AEP, Downe S, et al. From grief, guilt pain and stigma to hope and pride -- a systematic review and meta-analysis of mixed-method research of the psychosocial impact of stillbirth. BMC Pregnancy Childbirth [Internet]. 2016;16(1):9. Available from: http://dx.doi.org/10.1186/s12884-016-0800-8
4. Sarkar A, Siwatch S, Aggarwal N, Singla R, Grover S. The unheard parental cry of a stillbirth: fathers and mothers. Arch Gynecol Obstet [Internet]. 2021; Available from: https://doi.org/10.1007/s00404-021-06120-9
5. Black M, Shetty A, Bhattacharya S. Obstetric outcomes subsequent to intrauterine death in the first pregnancy. Bjog [Internet]. 2008;115. Available from: http://dx.doi.org/10.1111/j.1471-0528.2007.01562.x
6. Lamont K, Scott NW, Jones GT, Bhattacharya S. Risk of recurrent stillbirth: systematic review and meta-analysis. BMJ (Clinical Res Ed [Internet]. 2015;350(8014):h3080. Available from: http://www.hlisd.org/LibraryDetail.aspx?LibraryID=3872
7. Hughes P, Turton P, Hopper E, McGauley GA, Fonagy P. Disorganised attachment behaviour among infants born subsequent to stillbirth. J Child Psychol Psychiatry [Internet]. 2001;42(6):791–801. Available from: http://www.hlisd.org/LibraryDetail.aspx?LibraryID=3872
8. Mills TA, Ricklesford C, Cooke A, Heazell AEP, Whitworth M, Lavender T. Parents' experiences and expectations of care in pregnancy after stillbirth or neonatal death: a metasynthesis. BJOG [Internet]. 2014;121. Available from: http://dx.doi.org/10.1111/1471-0528.12656
9. Burden C, Bakhbakhi D, Heazell AE, Lynch M, Timlin L, Bevan C, et al. Parents' Active Role and ENgagement in The review of their Stillbirth/perinatal death 2 (PARENTS 2) study: a mixed-methods

For peer review only - http://bmjopen.bmj.com/site/about/guidelines.xhtml

study of implementation. BMJ Open [Internet]. 2021 Mar 1;11(3):e044563. Available from: http://bmjopen.bmj.com/content/11/3/e044563.abstract
10. Bakhbakhi D, Siassakos D, Storey C, Heazell A, Lynch M, Timlin L, et al. PARENTS 2 study protocol: pilot of Parents{\textquoteright} Active Role and ENgagement in the review of Their Stillbirth/perinatal death. BMJ Open [Internet]. 2018;8(1). Available from: https://bmjopen.bmj.com/content/8/1/e020164
11. Bakhbakhi D, Burden C, Storey C, Heazell AE, Lynch M, Timlin L, et al. PARENTS 2 Study: a qualitative study of the views of healthcare professionals and stakeholders on parental engagement in the perinatal mortality review{\textemdash}from {\textquoteleft}bottom of the pile{\textquoteright} to joint learning. BMJ Open [Internet]. 2018;8(11). Available from: https://bmjopen.bmj.com/content/8/11/e023792
12. Bakhbakhi D, Siassakos D, Burden C, Jones F, Yoward F, Redshaw M, et al. Learning from deaths: Parents' Active Role and ENgagement in The review of their Stillbirth/perinatal death (the PARENTS 1 study). BMC Pregnancy Childbirth. 2017 Oct;17(1):333.
13. Siassakos D, Jackson S, Gleeson K, Chebsey C, Ellis A, Storey C. All bereaved parents are entitled to good care after stillbirth: a mixed-methods multicentre study (INSIGHT). BJOG [Internet]. 2018;125(2):160. Available from: http://ovidsp.ovid.com/ovidweb.cgi?T=JS&PAGE=fulltext&MODE=ovid&CSC=Y&NEWS=N&D=ovft&SEARCH=1470-0328.is+and+%22125%22.vo+and+%222%22.ip+and+%22160%22.pg
14. Ladhani NNN, Fockler ME, Stephens L, Barrett JFR, Heazell AEP. No. 369-Management of Pregnancy Subsequent to Stillbirth. J Obstet Gynaecol Canada [Internet]. 2018 Dec 1;40(12):1669–83. Available from: https://doi.org/10.1016/j.jogc.2018.07.002
15. Koopmans L, Wilson T, Cacciatore J, Flenady V. Support for mothers, fathers and families after perinatal death. Cochrane Database Syst Rev [Internet]. 2013;(6). Available from: http://dx.doi.org/10.1002/14651858.CD000452.pub3
16. Gynaecologists. RC of O and. Each Baby Counts: 2015 Summary Report.
17. Wojcieszek AM, Shepherd E, Middleton P, Lassi ZS, Wilson T, Murphy MM, et al. Care prior to and during subsequent pregnancies following stillbirth for improving outcomes. Cochrane Database Syst Rev [Internet]. 2018;(12). Available from: https://doi.org//10.1002/14651858.CD012203.pub2
18. Hennegan JM, Henderson J, Redshaw M. Contact with the baby following stillbirth and parental mental health and well-being: a systematic review. BMJ Open [Internet]. 2015 Nov 1;5(11). Available from: http://bmjopen.bmj.com/content/5/11/e008616.abstract
19. Clarke M. Standardising outcomes for clinical trials and systematic reviews. Trials [Internet]. 2007;8(1):39. Available from: http://dx.doi.org/10.1186/1745-6215-8-39
20. Wojcieszek AM, Heazell AEP, Middleton P, Ellwood D, Silver RM, Flenady V. Research priorities and potential methodologies to inform care in subsequent pregnancies following stillbirth: a web-based survey of healthcare professionals, researchers and advocates. BMJ Open [Internet]. 2019 Jun 1;9(6):e028735. Available from: http://bmjopen.bmj.com/content/9/6/e028735.abstract
21. Williamson PR, Altman DG, Blazeby JM, Clarke M, Devane D, Gargon E, et al. Developing core outcome sets for clinical trials: issues to consider. Trials [Internet]. 2012;13(1):132. Available from: http://dx.doi.org/10.1186/1745-6215-13-132
22. Initiative C. COMET [Internet]. [cited 2017 Jan 9]. Available from: http://www.comet-initiative.org/
23. Williamson PR, Altman DG, Bagley H, Barnes KL, Blazeby JM, Brookes ST, et al. The COMET Handbook: version 1.0. Trials [Internet]. 2017 Jun;18(3):280. Available from: https://doi.org/10.1186/s13063-017-1978-4
24. Gorst SL, Gargon E, Clarke M, Blazeby JM, Altman DG, Williamson PR. Choosing Important Health Outcomes for Comparative Effectiveness Research: An Updated Review and User Survey. PLoS One [Internet]. 2016 Jan 19;11(1):e0146444. Available from: http://dx.doi.org/10.1371%2Fjournal.pone.0146444

For peer review only - http://bmjopen.bmj.com/site/about/guidelines.xhtml

25. Boers M, Kirwan JR, Wells G, Beaton D, Gossec L, d'Agostino M-A, et al. Developing Core Outcome Measurement Sets for Clinical Trials: OMERACT Filter 2.0. J Clin Epidemiol [Internet]. 2017 Jan 9;67(7):745–53. Available from: http://dx.doi.org/10.1016/j.jclinepi.2013.11.013
26. Duffy JMN, van 't Hooft J, Gale C, Brown M, Grobman W, Fitzpatrick R, et al. A protocol for developing, disseminating, and implementing a core outcome set for pre-eclampsia. Pregnancy Hypertens An Int J Women's Cardiovasc Heal [Internet]. 2016;6(4):274–8. Available from: http://www.sciencedirect.com/science/article/pii/S2210778916300149
27. Potter S, Davies C, Holcombe C, Weiler-Mithoff E, Skillman J, Vidya R, et al. International development and implementation of a core measurement set for research and audit studies in implant-based breast reconstruction: a study protocol. BMJ Open [Internet]. 2020 Jan 1;10(1):e035505. Available from: http://bmjopen.bmj.com/content/10/1/e035505.abstract
28. Khalil A, Perry H, Duffy J, Reed K, Baschat A, Deprest J, et al. Twin–Twin Transfusion Syndrome: study protocol for developing, disseminating, and implementing a core outcome set. Trials [Internet]. 2017;18(1):325. Available from: https://doi.org/10.1186/s13063-017-2042-0
29. Webbe J, Brunton G, Ali S, Duffy JM, Modi N, Gale C. Developing, implementing and disseminating a core outcome set for neonatal medicine. BMJ Paediatr open [Internet]. 2017 Jul 26;1(1):e000048–e000048. Available from: https://pubmed.ncbi.nlm.nih.gov/29637104
30. Hirsch M, Duffy JMN, Kusznir JO, Davis CJ, Plana MN, Khan KS, et al. Variation in outcome reporting in endometriosis trials: a systematic review. Am J Obstet Gynecol [Internet]. 2016 Apr 1;214(4):452–64. Available from: https://doi.org/10.1016/j.ajog.2015.12.039
31. Kirkham JJ, Davis K, Altman DG, Blazeby JM, Clarke M, Tunis S, et al. Core Outcome Set-STAndards for Development: The COS-STAD recommendations. PLOS Med [Internet]. 2017 Nov 16;14(11):e1002447. Available from: https://doi.org/10.1371/journal.pmed.1002447
32. Kirkham JJ, Gorst S, Altman DG, Blazeby JM, Clarke M, Tunis S, et al. Core Outcome Set-STAndardised Protocol Items: the COS-STAP Statement. Trials [Internet]. 2019;20(1):116. Available from: https://doi.org/10.1186/s13063-019-3230-x
33. Kirkham JJ, Gorst S, Altman DG, Blazeby JM, Clarke M, Devane D, et al. Core Outcome Set–STAndards for Reporting: The COS-STAR Statement. PLOS Med [Internet]. 2016 Oct 18;13(10):e1002148. Available from: http://dx.doi.org/10.1371%2Fjournal.pmed.1002148
34. Dodd S, Harman N, Taske N, Minchin M, Tan T, Williamson PR. Core outcome sets through the healthcare ecosystem: the case of type 2 diabetes mellitus. Trials [Internet]. 2020;21(1):570. Available from: https://doi.org/10.1186/s13063-020-04403-1
35. Innovation VH. Covidence systematic review software. Melbourne, Australia;
36. Satement P. Preferred Reporting Items for Systematic Reviews and Meta-Analyses (PRISMA).
37. de Wit M, Abma T, Koelewijn-van Loon M, Collins S, Kirwan J. Involving patient research partners has a significant impact on outcomes research: a responsive evaluation of the international OMERACT conferences. BMJ Open [Internet]. 2013 Jan 1;3(5). Available from: http://bmjopen.bmj.com/content/3/5/e002241.abstract
38. Smith LK, Dickens J, Bender Atik R, Bevan C, Fisher J, Hinton L. Parents' experiences of care following the loss of a baby at the margins between miscarriage, stillbirth and neonatal death: a UK qualitative study. BJOG An Int J Obstet Gynaecol [Internet]. 2020 Jun 1;127(7):868–74. Available from: https://doi.org/10.1111/1471-0528.16113
39. Baker, S and Edwards R. How many qualitative interviews is enough? [Internet]. National Centre for Research Methods. [cited 2017 Jan 15]. Available from: http://eprints.ncrm.ac.uk/2273/4/how_many_interviews.pdf
40. Ziebland S, McPherson A. Making sense of qualitative data analysis: an introduction with illustrations from DIPEx (personal experiences of health and illness). Med Educ [Internet]. 2006 May 1;40(5):405–14. Available from: http://dx.doi.org/10.1111/j.1365-2929.2006.02467.x

For peer review only - http://bmjopen.bmj.com/site/about/guidelines.xhtml

41. Tong A, Sainsbury P, Craig J. Consolidated criteria for reporting qualitative research (COREQ): a 32-item checklist for interviews and focus groups. Int J Qual Heal Care [Internet]. 2007 Nov 19;19(6):349 LP – 357. Available from: http://intqhc.oxfordjournals.org/content/19/6/349.abstract
42. Dodd S, Clarke M, Becker L, Mavergames C, Fish R, Williamson PR. A taxonomy has been developed for outcomes in medical research to help improve knowledge discovery. J Clin Epidemiol [Internet]. 2017/12/28. 2018 Apr;96:84–92. Available from: https://pubmed.ncbi.nlm.nih.gov/29288712
43. Brookes ST, Chalmers KA, Avery KNL, Coulman K, Blazeby JM, Barham CP, et al. Impact of question order on prioritisation of outcomes in the development of a core outcome set: a randomised controlled trial. Trials [Internet]. 2018;19(1):66. Available from: https://doi.org/10.1186/s13063-017-2405-6
44. Drennan J. Cognitive interviewing: verbal data in the design and pretesting of questionnaires. J Adv Nurs [Internet]. 2003 Apr 1;42(1):57–63. Available from: https://doi.org/10.1046/j.1365-2648.2003.02579.x
45. Collins D. Pretesting survey instruments: An overview of cognitive methods. Qual Life Res [Internet]. 2003;12(3):229–38. Available from: https://doi.org/10.1023/A:1023254226592
46. Mathioudakis AG, Abroug F, Agusti A, Bakke P, Bartziokas K, Beghe B, et al. Core outcome set for the management of acute exacerbations of chronic obstructive pulmonary disease: the COS-AECOPD ERS Task Force study protocol. ERJ Open Res [Internet]. 2020 Jul 1;6(3):193–2020. Available from: http://openres.ersjournals.com/content/6/3/00193-2020.abstract
47. Srikandarajah N, Noble A, Clark S, Wilby M, Freeman BJC, Fehlings MG, et al. Cauda Equina Syndrome Core Outcome Set (CESCOS): An international patient and healthcare professional consensus for research studies. PLoS One [Internet]. 2020 Jan 10;15(1):e0225907–e0225907. Available from: https://pubmed.ncbi.nlm.nih.gov/31923259
48. Hagen NA, Stiles C, Nekolaichuk C, Biondo P, Carlson LE, Fisher K, et al. The Alberta Breakthrough Pain Assessment Tool for Cancer Patients: A Validation Study Using a Delphi Process and Patient Think-Aloud Interviews. J Pain Symptom Manage [Internet]. 2008;35(2):136–52. Available from: http://www.sciencedirect.com/science/article/pii/S088539240700677X
49. Willis GB. Cognitive interviewing: a tool for improving questionnaire design [Internet]. Thousand Oaks: Sage; 2005. Available from: https://doi.org/10.4135/9781412983655
50. French DP, Cooke R, Mclean N, Williams M, Sutton S. What Do People Think about When They Answer Theory of Planned Behaviour Questionnaires?: A `Think Aloud' Study. J Health Psychol [Internet]. 2007 Jul 1;12(4):672–87. Available from: https://doi.org/10.1177/1359105307078174
51. Bradbury K, Morton K, Band R, van Woezik A, Grist R, McManus RJ, et al. Using the Person-Based Approach to optimise a digital intervention for the management of hypertension. PLoS One [Internet]. 2018 May 3;13(5):e0196868. Available from: https://doi.org/10.1371/journal.pone.0196868
52. Yardley L, Ainsworth B, Arden-Close E, Muller I. The person-based approach to enhancing the acceptability and feasibility of interventions. Pilot Feasibility Stud [Internet]. 2015;1(1):37. Available from: https://doi.org/10.1186/s40814-015-0033-z
53. Calvert M, Blazeby J, DG A, al et. Reporting of patient-reported outcomes in randomized trials: The consort pro extension. JAMA [Internet]. 2013 Feb 27;309(8):814–22. Available from: http://dx.doi.org/10.1001/jama.2013.879
54. Gargon E, Crew R, Burnside G, Williamson PR. Higher number of items associated with significantly lower response rates in COS Delphi surveys. J Clin Epidemiol [Internet]. 2019;108:110–20. Available from: http://www.sciencedirect.com/science/article/pii/S0895435618307765
55. Hall DA, Smith H, Heffernan E, Fackrell K, Group for the COM in TID (COMiT'ID) RS. Recruiting and retaining participants in e-Delphi surveys for core outcome set development: Evaluating the COMiT'ID study. PLoS One [Internet]. 2018 Jul 30;13(7):e0201378. Available from: https://doi.org/10.1371/journal.pone.0201378
56. Alkhaffaf B, Blazeby JM, Metryka A, Glenny A-M, Adeyeye A, Costa PM, et al. Methods for conducting international Delphi surveys to optimise global participation in core outcome set development: a case

For peer review only - http://bmjopen.bmj.com/site/about/guidelines.xhtml

study in gastric cancer informed by a comprehensive literature review. Trials [Internet]. 2021;22(1):410. Available from: https://doi.org/10.1186/s13063-021-05338-x

57. Harris PA, Taylor R, Thielke R, Payne J, Gonzalez N, Conde JG. Research electronic data capture (REDCap)—A metadata-driven methodology and workflow process for providing translational research informatics support. J Biomed Inform [Internet]. 2009;42(2):377–81. Available from: http://www.sciencedirect.com/science/article/pii/S1532046408001226

58. What is task sharing? Review of WHO Guidelines and ongoing research on task sharing of contraceptive services. Eur J Contracept Reprod Heal Care [Internet]. 2016;21(SUPPL. 1):11. Available from: http://ovidsp.ovid.com/ovidweb.cgi?T=JS&PAGE=reference&D=emed18&NEWS=N&AN=72324861

59. Bakhbakhi D, Siassakos D, Lynch M, Timlin L, Storey C, Heazell A, et al. PARENTS 2 Study: consensus report for parental engagement in the perinatal mortality review process. Ultrasound Obstet Gynecol [Internet]. 2018 Oct 7;0(ja). Available from: https://doi.org/10.1002/uog.20139

60. Harvey N, Holmes CA. Nominal group technique: An effective method for obtaining group consensus. Int J Nurs Pract [Internet]. 2012 Apr 1;18(2):188–94. Available from: https://doi.org/10.1111/j.1440-172X.2012.02017.x

61. Perry H, Duffy JMN, Umadia O, Khalil A. Outcome reporting across randomized trials and observational studies evaluating treatments for twin–twin transfusion syndrome: systematic review. Ultrasound Obstet Gynecol [Internet]. 2018;52(5):577–85. Available from: https://obgyn.onlinelibrary.wiley.com/doi/abs/10.1002/uog.19068

62. Duffy JMN, AlAhwany H, Bhattacharya S, Collura B, Curtis C, Evers JLH, et al. Developing a core outcome set for future infertility research: an international consensus development study†‡. Hum Reprod [Internet]. 2020 Dec 1;35(12):2725–34. Available from: https://doi.org/10.1093/humrep/deaa241

63. Hsieh H-F, Shannon SE. Three Approaches to Qualitative Content Analysis. Qual Health Res [Internet]. 2005 Nov 1;15(9):1277–88. Available from: https://doi.org/10.1177/1049732305276687

For peer review only

For peer review only - http://bmjopen.bmj.com/site/about/guidelines.xhtml

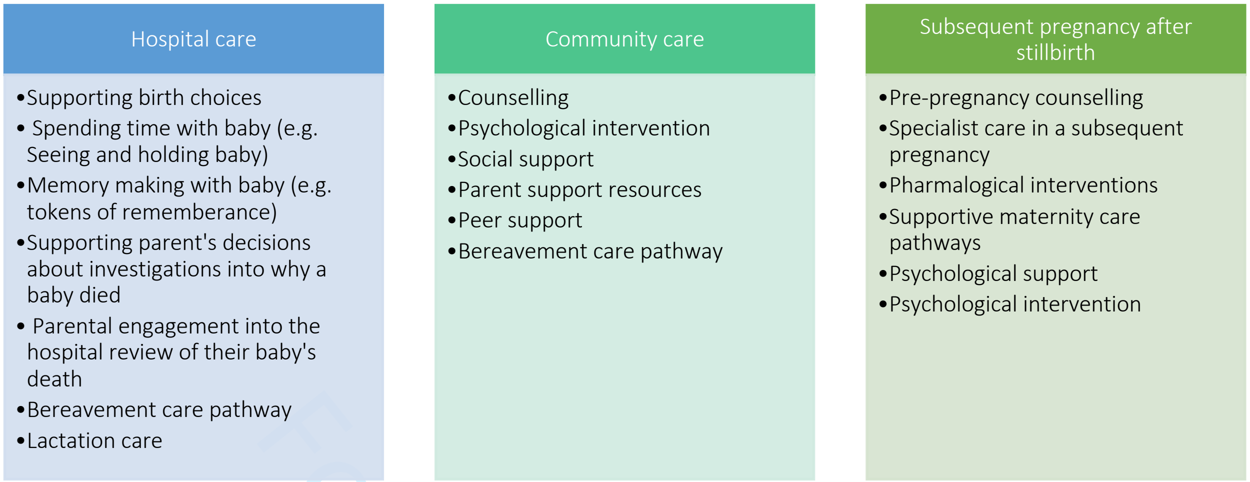

Figure 1: Types of interventions after stillbirth that should be evaluated using outcomes identified in the core outcome set

For peer review only

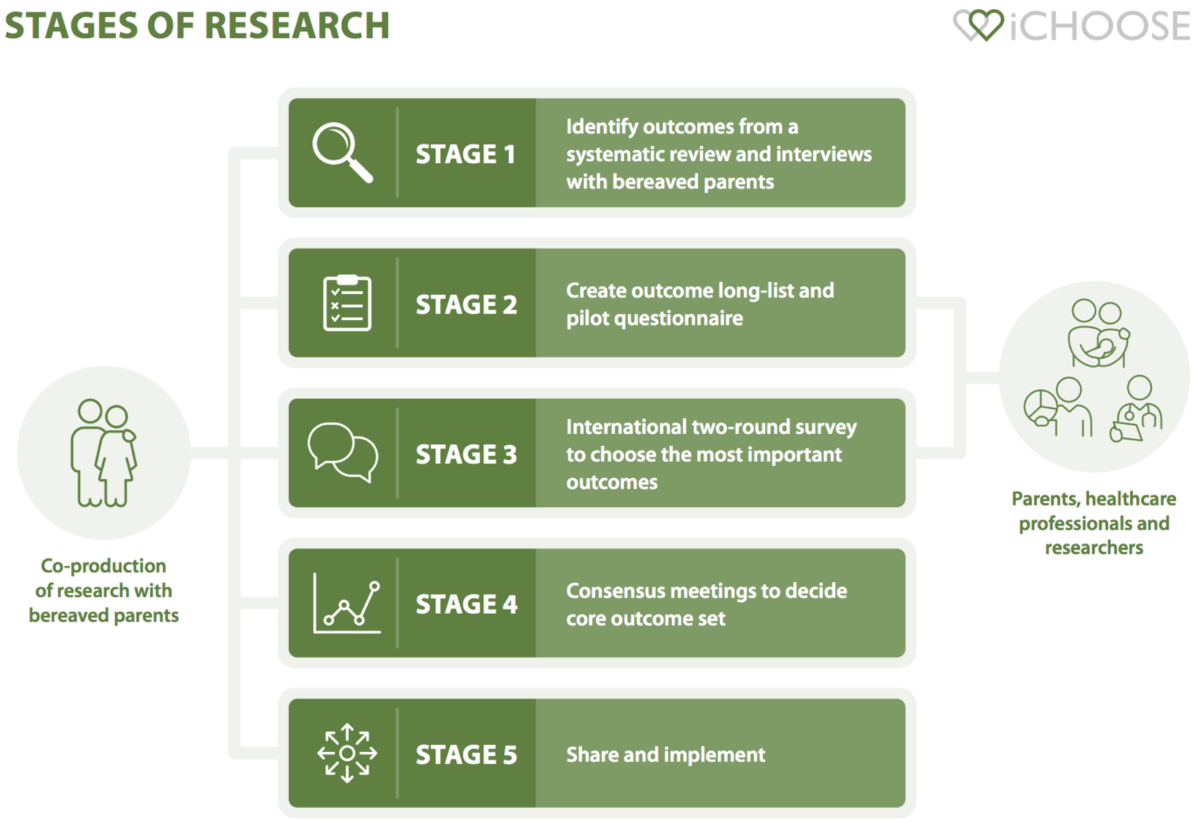

Figure 2: iCHOOSE Study overview

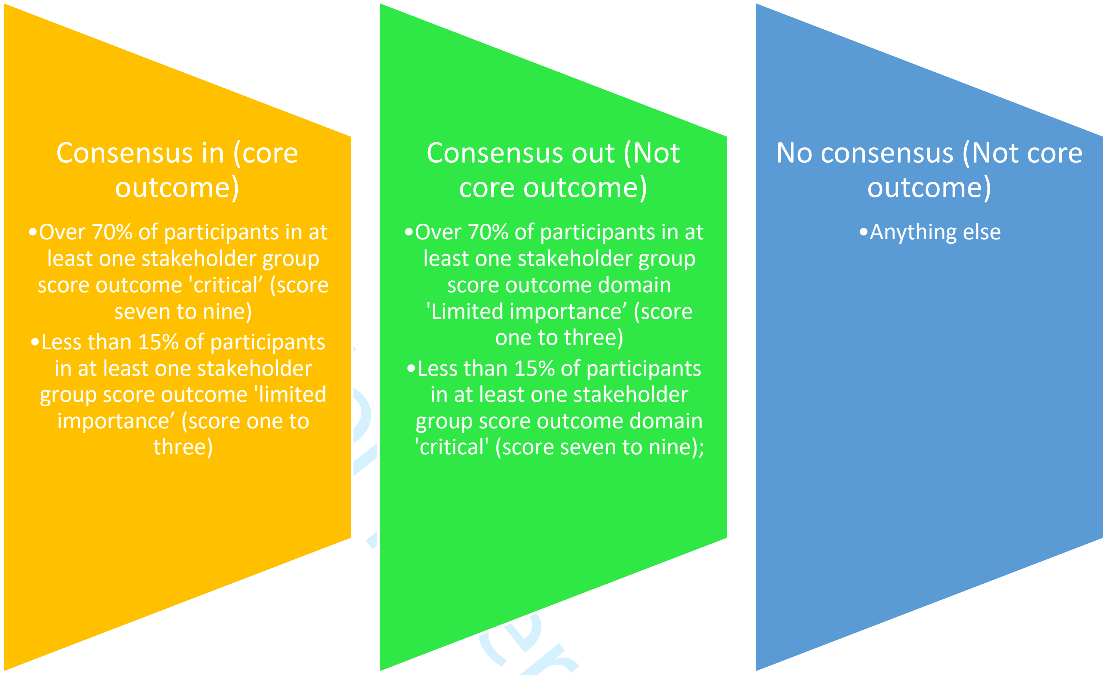

Figure 3: Consensus Definition

For peer review only - http://bmjopen.bmj.com/site/about/guidelines.xhtml

Core Outcome Set-STAndardised Protocol Items (COS-STAP) Checklist for the iCHOOSE Study

Item Number	Name	Location
1	Identify in the title that the paper describes the protocol for the planned development of a COS	TITLE/ABSTRACT<br>Page 1
2	Provide a structured abstract	ABSTRACT<br>Page 1-2
3	Describe the background and explain the rationale for developing the COS	INTRODUCTION<br>Page 3-4
P	Describe the specific objectives with reference to developing a COS	INTRODUCTION and AIMS AND OBJECTIVES<br>Page 4
5	Describe the health condition(s) and population(s) that will be covered by the COS	INTRODUCTION AND METHODS AND ANALYSIS<br>Page 5<br><br>[Scope – Health Condition and Population]
6	Describe the intervention(s) that will be covered by the COS	INTRODUCTION AND METHODS AND ANALYSIS<br>[Scope – Intervention]<br>Page 5
7	Describe the setting(s) that will be covered by the COS	INTRODUCTION AND METHODS AND ANALYSIS<br>Page 5
8	Indicate the COS study registration details and registry name. If not yet registered indicate the intended registry	REGISTRATION DETAILS<br>Page 2
9	Describe any study oversight committees	STEERING COMMITTEE AND PATIENT AND PUBLIC INVOLVEMENT<br>Page 5 & 6
10	Describe sources of funding, role of funders	FUNDING STATEMENT Page 13-14
11	Describe any potential conflicts of interest within the study team and how these will be managed	CONFLICTS OF INTEREST<br>Page 14
12	Describe the stakeholder groups to be involved in the COS development process and the rationale for their involvement	METHODS –STAKEHOLDERS<br>Page 9-10

For peer review only

13	Describe the eligibility criteria for individuals from each stakeholder group	METHODS –STAKEHOLDERS Page 9-10
14	Describe how individuals of each stakeholder groups will be identified	METHODS –STAKEHOLDERS Page 9-10
15	Describe how individuals of each stakeholder group will be chosen from within the stakeholder group	METHODS –STAKEHOLDERS Page 9-10
16	Describe how many planned individuals within each stakeholder group will be invited to participate in the consensus process	METHODS –STAKEHOLDERS [Participants – Sample Size] Page 10
17	Describe how individuals will be invited to take part in the consensus process	METHODS –STAKEHOLDERS Page 10
18	Describe the information sources that will be used to identify the list of outcomes. Outline the methods or reference other protocols/papers.	METHODS – SYSTEMATIC REVIEW AND QUALITATIVE INTERVIEWS Page 6&7
19	Describe how outcomes may be dropped/combined, with reasons	METHODS Page 11
20	Describe the methods to identify outcome descriptor terms	METHODS Page 8&9
21	Describe the plans for how the consensus process will be undertaken	METHODS Page 9-12
22	Describe what information will be presented to participants at the start of the consensus process	METHODS Page 8-9
23	Describe what each participant will be asked to do at each stage of the consensus process	METHODS Page 11
24	Describe how the participants will receive any feedback during the consensus process	METHODS Page 11
25	Describe how non-response (or partial response) will be handled during the consensus process	METHODS Page 11
26	Describe how the study material will be made patient friendly and understandable (if relevant)	METHODS Page 8-9
27	Describe the consensus definition	METHODS Page 11-12
28	Describe the procedure for determining how outcomes will be added/combined/dropped from consideration during the consensus process	METHODS Page 11
29	Describe how outcomes will be scored and summarised	METHODS Page 11

For peer review only

30	Describe how the response rate will be maximised	METHODS<br>Page 10
31	Describe how attrition bias will be assessed	METHODS<br>Page 11
32	Describe any software that will be used during the consensus process and to analyse the results	METHODS<br>Page 10
33	Describe any plans for obtaining research ethics committee / institutional review board approval in relation to the consensus process (if relevant)	ETHICS/ DISSEMINATION<br>Page 13
34	Describe how informed consent will be obtained (if relevant)	ETHICS/ DISSEMINATION & METHODS<br>Page 7,9, 10, 11,12
35	Describe any details about how the confidentiality of data collection will be preserved during the consensus process (if relevant)	METHODS<br>Page 10-11

For peer review only

A)The parent experience

I'd like you to tell me your story with as much detail as possible. Then I may have some extra questions if you have not covered them already. We would like to find out your experience and we are particularly interested in how your loss has affected you/and your partner's health.

1) The diagnosis of the stillbirth

- Can you take me back to the beginning of you story, perhaps start from when you first found out you were pregnant?

2) The time between diagnosis and birth (If applicable)

- What happened after you found out you your baby had died e.g. did you go home/stay in hospital/be induced?

3) The birth of your baby

- What was your experience of giving birth to your baby?
- How do you feel about the way you gave birth now?

4) Your stay in hospital

- What happened after your baby was born?

5) Memory Making

- How did you spend time with your baby? If you were unable to spend time with baby, then why not?

Examples include: did you see or hold the baby, did you take photos, foot prints

- How did it make you feel at the time?
- What do you feel about the experience now?
- Which memories are the most meaningful to you now?

6) Post mortem & hospital tests

- What hospital tests did you or your baby have afterwards?
- What information did you learn from the post mortem and/or additional tests?
- How did you feel about your choice at the time & how do you feel about it now? (If did or did not have PM)

7) The review process by the hospital (perinatal mortality review)

- What was your experience of the hospital review process of you and your baby's case?
- Were the involved in the case?
- If parental engagement In review – how did it affect them?

8) The funeral

- What information & support were you given about the baby's funeral?
- If had funeral, can you tell me more about it
- How did that make you feel at the time/how do you feel about it now?

9) When you went home

- Can you tell me about how you made the decision about going home?
- What happened in the days/weeks after you went home?
- How did you feel when you went home?

10) Follow up care by healthcare professionals (e.g hospital consultant, midwife, GP, anyone else?)

For peer review only

- What follow up care did you receive from the hospital e.g. follow up appointment with consultant, bereavement midwife?
- What advice were you given in your follow up appointment about becoming pregnant again?
  - *Were you told how long you should wait before becoming pregnant again (if not discussed)? What will the care be like? What will the birth be like? What were your thoughts about this?*
- What was the contact with the GP/community midwife like?
- What was the impact of the healthcare professionals care on you?

11) Additional care

- Following your experience did you seek any further professional advice or care?
  - *Examples include second opinion, counselling*
- If yes, how did you feel about this care?
- What was the impact of counselling on you? May consider benefits and harms?
- Were there any other ways/methods you dealt with the stillbirth?
  - *Examples could include exercise, mindfulness, charity work, yoga, new hobby, support groups*

12) Support groups

- Did you seek any help from support groups?
- How did that impact you?
- Did you seek any support from online communities?

13) Plans for future pregnancies

- What were your thoughts about becoming pregnant again?

What influenced your decision? Did you seek any alternative advice? What were the sources of your information?

B) Outcomes

1a) Impact on next pregnancy (if applicable)

- Have you had any more pregnancies since? Tell me about your next pregnancy
- How did your previous experience affect your next pregnancy?
- How were you looked after in your next pregnancy?
- Examples: were you treated differently by medical professionals during antenatal care? did you have any extra care, appointments, scans or tests? Was the type of birth different?
- How did becoming pregnant affect you and/or your partner?
- How did being pregnant affect your health? (physical & mental)
- What support (psychological) was offered during pregnancy and after birth?
- Were there any complications in the subsequent pregnancy?
- Did the subsequent pregnancy have an effect on other areas of your life?

1b) If no further pregnancies:

- How did not becoming pregnant again affect you?
- Did you seek any fertility treatment & If yes, could you tell me about it and how did it effect you?

2) Impact of experience on physical & mental health

Opening statement: In this part of interview I will ask about you and your partners health including the impact the stillbirth had on your physical and mental health

- How do you think your experience affected your health in the short term (physical and mental)?
- How do you think your experience affected your health in the long term (physical and mental)?

For peer review only

If does not mention:

Has your experience affected your mental health?
Have you had low mood/anxiety/PTSD?
How has your experience affected your physical health?
Has is it affected your sleep?
Has your experience affected your body image/self-esteem?

- Did you do anything to improve your health after the experience? What were you encouraged to do?
- Could there have been anything done to minimise the impact on you and your health?
- Have any new medical conditions emerged since the stillbirth? Have you had any treatment/therapy?
- What medications have you taken (if any) following the stillbirth?

14) Relationships

- How has your experience affected your relationships?
  - Your partner (weeks, months, years); Your family (weeks, months, years); Your friends (weeks, months, years)
- What was the impact of new babies on you?

15) Relationship with children (if applicable)

- How has your experience affected your relationship with your children?
- Has your experience affected bonding/parenting with your children?
- Have your children needed to have any additional support?

16) Communication

- How did you tell people about your stillbirth?
- How did they respond and how did it affect you?
- What was the impact of social media/media/news stories on you?

17) Employment - going back to work/Finances

- How did you break the news to your colleagues?
- How did your experience affect your job? (if applicable)
- How did the response of your employer affect you (and/or your decision to return to work)?
- How did your experience affect your finances? How did finances affect your decision making?
- If money was no issue, when would have been the right time to return to work in your opinion?

18) Outcomes to develop core outcome set

- If we were going to improve care or research after stillbirth what would be important in your opinion to measure to see if the care worked? Examples include mental health, physical, return to work
- If we were going to improve health after stillbirth what aspect of health would be important to you to improve?
- If unable to answer: How would you improve about the care you received. If that care was improved how would that affected your life and health?

19) Key messages to parents & healthcare professionals

Opening statement: Bearing In mind parents might receive this information whilst in hospital/preparing to go into hospital or newly discharged…

- What would be your advice to parent who might be going through a similar experience?

- What would be your overall key messages to healthcare professionals looking after parents who experience stillbirth?

For peer review only

For peer review only

Supplementary material 3: Think aloud topic guide

Interview Guide

Harmonising outcomes for research and care after stillbirth (The iCHOOSE Study)

Think-aloud interviews for the development of a core outcome set questionnaire

Introduction to study

These interviews aim to find out about how you go about completing a questionnaire to develop a core outcome set. You will be asked to provide a running commentary on how you rate the importance of individual outcomes. We are particularly interested in how you understand the questions, how difficult or easy it is to rate the outcomes, the wording of the outcomes and any changes you would make to the questionnaire. We are also interested in whether there are additional outcomes that should be included in the questionnaire.

If at any time you don't want to continue the interview, you are free to tell me that you wish to stop, and we can either take a break or you can stop completely. It's fine for you to do that.

Interview Instructions

Participants will be read the following instructions, adapted from Green and Gilhooly (1996) and French et al. (2007):

We are interested in how people complete the following questionnaire. This questionnaire will ask you to rank outcomes on how important they are to you to measure in research and evaluating care after stillbirth. We want to check that people understand the questions in the way that we meant them. To do this, I am going to ask you to 'think aloud' as you complete the questionnaire. What I mean by 'think aloud' is that I want you to tell me everything you are thinking as you read each question and decide how to answer it. I would like you to talk aloud constantly. I don't want you to plan out what you say or try to explain to me what you are saying. Just act as if you are alone in the room speaking to yourself. If you are silent for any long period of time, I will ask you to talk or ask you question to help you. Please try to speak as clearly as possible, as I shall be recording you as you speak. Do you understand what I want you to do?

Interview question probes (Adapted from Collins (2003), French et al (2007) and McCorry (2013))

General

- Tell me what are you thinking?
- How did you go about rating that outcome?
- How easy or difficult did you find this outcome to rate?
- Would you like to make any changes to this question/outcome?

For peer review only

Comprehension

- What does that outcome mean to you?
- What did you understand by this outcome?
- Are there any problems with the wording of this outcome?

Retrieval

- How did you calculate your answer?
- Is this outcome relevant to a particular time period that you can relate to?
- How applicable is this outcome to your individual circumstances?

Confidence judgement

- How sure of your answer are you?

Response

- How did you feel about answering this question?

Additional outcomes

- Would you like to include any other additional outcomes?

For peer review only

Supplementary material 4: Table of changes for think aloud interviews and questionnaire development

Delphi Questionnaire for core outcome set for research after stillbirth					
Negative Comments	*Positive Comments*	*Possible Change*	*Reason for change*	*Agreed change*	*MoScoW*

For peer review only

For peer review only
