# Supplementary material for: Protocol for the development of a core outcome set for stillbirth care research (iCHOOSE Study)
Source: BMJ Open. 2022 Feb 9;12(2):e056629. doi: 10.1136/bmjopen-2021-056629 (PMC8830254; doi:10.1136/bmjopen-2021-056629)
Supplement: Reviewer comments [file bmjopen-2021-056629.reviewer_comments.pdf]

PEER REVIEW HISTORY

BMJ Open publishes all reviews undertaken for accepted manuscripts. Reviewers are asked to complete a checklist review form (**http://bmjopen.bmj.com/site/about/resources/checklist.pdf**) and are provided with free text boxes to elaborate on their assessment. These free text comments are reproduced below.

ARTICLE DETAILS

**TITLE (PROVISIONAL)**	Protocol for the development of a core outcome set for stillbirth care research (iCHOOSE Study)
**AUTHORS**	Bakhbakhi, Danya; Fraser, Abigail; Siasakos, Dimitris; Hinton, Lisa; Davies, Anna; Merriel, Abi; Duffy, James; Redshaw, Maggie; Lynch, Mary; Timlin, Laura; Flenady, Vicki; Heazell, Alexander; Downe, Soo; Slade, Pauline; Brookes, Sara; Wojcieszek, Aleena; Murphy, Margaret; de Oliveira Salgado, Heloisa; Pollock, Danielle; Aggarwal, Neelam; Attachie, Irene; Leisher, Susannah; Kihusa, Wanijiru; Mulley, Kate; Wimmer, Lindsey; ., UK iCHOOSE parent involvement group; Burden, Christy

This article was not externally reviewed at BMJ Open. Protocols that have been independently assessed prior to submission to BMJ Open are usually fast-tracked to publication on the grounds that further substantial changes will not be possible. This independent assessment will usually be external, independent review for both a substantial grant award from a non-commercial or government funder and ethics approval.
